# Composition and Genetic Diversity of the *Nicotiana tabacum* Microbiome in Different Topographic Areas and Growth Periods

**DOI:** 10.3390/ijms19113421

**Published:** 2018-10-31

**Authors:** Xiao-Long Yuan, Min Cao, Xin-Min Liu, Yong-Mei Du, Guo-Ming Shen, Zhong-Feng Zhang, Jin-Hai Li, Peng Zhang

**Affiliations:** 1 Tobacco Research Institute of Chinese Academy of Agricultural Sciences, Qingdao 266101, China; rayrock@126.com (X.-L.Y.); liuxinmin@caas.cn (X.-M.L.); duyongmei@caas.cn (Y.-M.D.); shenguoming@caas.cn (G.-M.S.); zhangzhongfeng@caas.cn (Z.-F.Z.); 2 College of Marine Life Sciences, Ocean University of China, Qingdao 266100, China; caominjiayou@163.com; 3 National Tobacco Corporation in Hubei province, Wuhan 430000, China; lijinhai0532@gmail.com

**Keywords:** *Nicotiana tabacum*, topographic areas, growth periods, fungal community composition, genetic diversity, ITS region

## Abstract

Fungal endophytes are the most ubiquitous plant symbionts on earth and are phylogenetically diverse. Studies on the fungal endophytes in tobacco have shown that they are widely distributed in the leaves, stems, and roots, and play important roles in the composition of the microbial ecosystem of tobacco. Herein, we analyzed and quantified the endophytic fungi of healthy tobacco leaves at the seedling stage (SS), resettling growth stage (RGS), fast-growing stage (FGS), and maturing stage (MS) at three altitudes (600, 1000, and 1300 m). We sequenced the internal transcribed spacer (ITS) region of fungal samples to delimit operational taxonomic units (OTUs) and phylogenetically characterize the communities. The results showed that the numbers of clustering OTUs at SS, RGS, FGS, and MS were 516, 709, 469, and 428, respectively. At the phylum level, species in Ascomycota and Basidiomycota had absolute predominance, representing 97.8% and 2.0% of the total number of species, respectively. We also found the number of fungi at the RGS and FGS stages was higher than those at the other two stages. Additionally, OTU richness was determined by calculating the Observed Species, Shannon, Simpson, Chao1, abundance-based coverage estimator (ACE), Good’s coverage and phylogenetic distance (PD)_whole_tree indices based on the total number of species. Our results showed RGS samples had the highest diversity indices. Furthermore, we found that the diversity of fungal communities tended to decrease with increasing altitude. The results from this study indicated that tobacco harbors an abundant and diverse endophytic fungal community, which provides new opportunities for exploring their potential utilization.

## 1. Introduction

Fungal endophytes are those microorganisms that live intercellularly or intracellularly in plants without causing apparent damage to host tissues [1]. There is a symbiotic relationship between endophytes and plant tissues, whereby plants can provide nutrition and protection for endophytes, and the endophytes in turn have significant influences on the plant, protecting their host from biotic and abiotic stresses [2]. Previously, endophytic fungi have been isolated from the healthy tissues of tobacco (*Nicotiana tabacum*) [3,4]. Studies on the fungal endophytes in tobacco have shown that they are widely distributed in the leaves, stems, and roots, and play important roles in the composition of the microbial ecosystem of tobacco [5]. Furthermore, these endophytes have various biological functions. Endophytic fungi in tobacco can not only promote the growth of tobacco and enhance their capacity to resist diseases, pests, and/or abiotic stresses (e.g., resistance to heavy metals and drought stress) but they also play positive roles in decreasing the nitrosamine contents in tobacco and improving the quality of tobacco leaves. Therefore, it is desirable to determine the species distribution and characteristics of endophytic fungi in tobacco through a comprehensive investigation at different growth stages and altitudes.

Traditionally, the taxonomic identification and classification of fungi have been performed based on their morphologies, growth conditions, and physiological and biochemical characteristics [6]. However, fungal taxa are complex, often lacking discriminatory morphological characters, and they are susceptible to environmental change. In earlier studies of the fungi associated with tobacco, researchers isolated fungi from tobacco leaves with diverse morphologies. Norse and Spurr found that *Alternaria*, *Penicillium*, *Aspergillus*, and *Cladosporium* spp. were frequent endophytes in *Nicotiana* spp. [3,5], whereas English and Mitchell found that *Penicillium*, *Aspergillus*, *Trichoderma*, and *Circnella* were the dominant populations [7,8]. Moreover, most species are not amenable to in vitro culturing [9]. Consequently, it is often difficult to identify species according to their morphologies. With the development of molecular biology techniques, DNA sequence analysis has been widely applied in the taxonomic identification of fungi. In this regard, the nuclear ribosomal internal transcribed spacer (ITS) region and 18s RDNA sequences are widely used markers [10,11,12,13]. The fungal ITS region includes the *ITS1*, *ITS2*, and *5.8S* genes. Typically, the *5.8S* gene is highly conserved compared with the ITS1 and ITS2 sequences. Thus, the ITS1 and ITS2 genes are generally used for resolution at the genus and species levels [14,15,16]. In 2011, Li et al. analyzed the community structure and diversity of fungi in tobacco at the mature stage of growth by amplifying the *18S* and *ITS* genes [4]. Although these previous studies undoubtedly laid the foundations for our study on the fungal communities of tobacco, these studies were neither systematic nor comprehensive, and it is therefore highly probable that information on certain uncultured fungi has been overlooked. Nowadays, high-throughput sequencing technologies can facilitate the identification of low abundance species and can reduce costs and have thus become the first choice for fungal community diversity analysis. Therefore, based on the characteristics of the ITS region, we can construct pair-end libraries and determine sequences using technologies such as the Illumina HiSeq sequencing platform. Furthermore, we can analyze the abundance of species by clustering operational taxonomic units (OTUs) and determining the differences among samples by analyzing their alpha and beta diversities.

In this study, we performed a metagenomic analysis of fungal communities at four growth stages (SS, RGS, FGS, and MS) of the healthy tobacco plants grown at three altitudes (600, 1000 and 1300 m) via high-throughput sequencing. Our main goal was to investigate the diversity and heritability of the *Nicotiana tabacum* microbiome. Specifically, we sought to compare the fungal communities at different growth stages, and examine the effects of altitudes on fungal community characteristics. The findings will not only provide a theoretical basis for the control of tobacco diseases, but also lay the foundations for future research on endophytic fungi in tobacco.

## 2. Results

### 2.1. Sequencing Results

Sequencing of the amplicon libraries representing four growth periods (SS, RGS, FGS, and MS stages) and three altitudes (600, 1000, and 1300 m) yielded a total of 2,888,484 raw reads before quality control (individual reads ranging from 70,606 to 89,979 bp). The average reads length was 313 bp before data processing. After quality control, the remaining high-quality reads in the dataset presented an average length of 314 bp (Table S1). We then assembled these high-quality reads into raw tags. Effective tags were finally obtained after qualification and removal of chimeras from raw tags. In order to study the species diversity of the sample, the effective tags of samples with more than 97% identity were clustered into OTUs.

### 2.2. Overview of Fungal Taxonomic Composition

**Figure 1:**
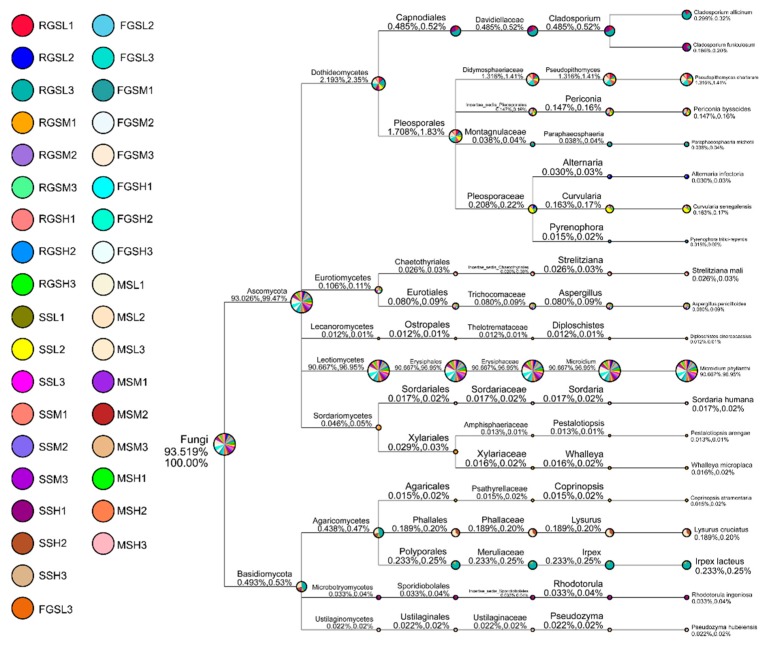
Taxonomic tree of all samples. Taxonomic tree of fungal communities of healthy tobacco plants at four growth stages (seedling (SS), resettling growth (RGS), fast-growing (FGS), and maturing (MS)).

For the annotation of all OTUs, we screened the identified species according to the classification results of each sample. At the phylum level, species in Ascomycota and Basidiomycota showed virtually absolute predominance, representing 97.8% and 2.0% of the total number of species, respectively. The top species at the class level were dominated by Leotiomycetes (90.74%), followed by Dothideomycetes (6.01%) and Agaricomycetes (0.85%). Erysiphales, Pleosporales, Capnodiales, Polyporales, and Agaricales, comprising approximately 90.71%, 4.97%, 0.58%, 0.27%, and 0.30%, respectively, of the samples, were the dominant groups at the order level. At the family level, Erysiphaceae accounted for 90.71% of the entire community, whereas Pleosporaceae, Davidiellaceae, Didymosphaeriaceae, Meruliaceae, and Leptosphaeriaceae taken together contributed to 5.19% of the total community in all samples. In terms of genera, the abundances of *Microidium*, *Cladosporium*, *Pseudopithomyces*, *Irpex*, and *Lysurus* were higher than those of other genera (Figure 1 and Table S2).

**Figure 2:**
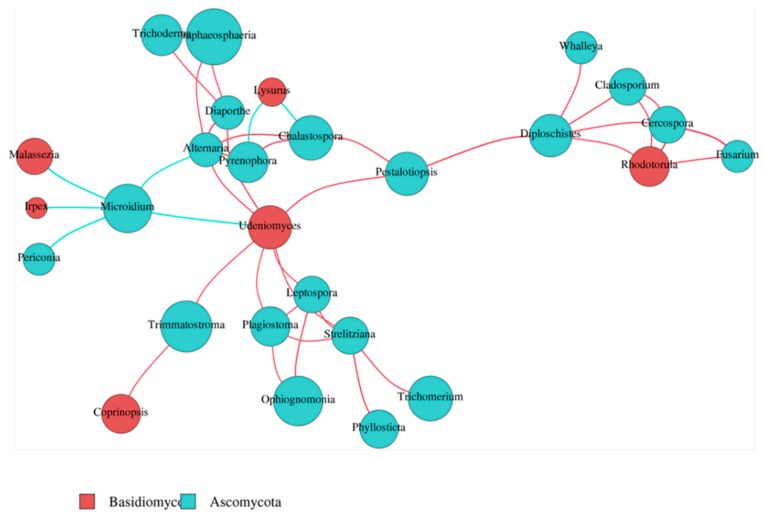
Network analysis at the genus level. Different nodes represent different genera, the node size represents the average relative abundance of the genus, and the nodes of the phylum have the same colors. There is a positive relationship between the thickness of the line and absolute correlation coefficient of species interaction. The blue line represents a negative correlation between the two genera, and the red line is a positive correlation.

Network analysis provides us with a new perspective for studying complex microbial community structures and functions. Given that relationships may vary under different environmental conditions, the advantage of dominant species and the species with close interactions can be directly determined [17]. Through analyzing the correlation index of all the sample calculations, with a cutoff = 0.6 to filter the absolute value of the correlation coefficient, coupled with species abundances, we constructed a figure showing the network of samples at the genus level. In our study, *Udeniomyces* and *Rhodotorula* in the phylum Basidiomycota were the genera with the highest abundances and were found to be located in the “hub” position of the network. All members of the *Ascomycota*, *Microidium, Trimmatostroma, Ophiognomonia, Trichomerium,* and *Paraphaeosphaeria* were the top five genera in terms of richness. Moreover, we found that *Udeniomyces* had a negative relationship with *Microidium* (Figure 2).

### 2.3. Dissimilarity of Fungal Communities at Different Growth Stages

**Figure 3:**
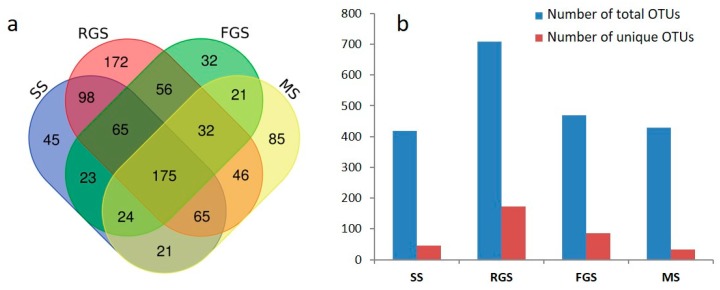
Comparison of fungal communities at different growth stages. (**a**) Venn diagram for samples among different combinations. Each ellipse represents a sample (group), with the number in the overlapping ellipses representing the number of operational taxonomic units (OTUs) shared between those samples (groups), and the number in the non-overlapping regions representing the number of unique OTUs of that sample (group). (**b**) Statistics of the OTUs number in different growth stages. Total OTUs refers to all the OTUs in a certain growth stage. Unique OTUs are those specific to a particular growth stage.

To understand the abundance of OTUs and the communities of fungi at different growth stages, we compared the richness of OTUs and species compositions at different growth stages. The number of clustering OTUs for stages SS, RGS, FGS, and MS were 516, 709, 469, and 428, respectively (Figure 3a,b). The results showed that the numbers of OTUs were highest in the RGS stages compared with those in the other three stages. Conversely, the number of OTUs in the MS was the lowest. Statistical analysis showed that there was a significant difference (*p* < 0.05) in the OTUs numbers in these four stages. Furthermore, we found that the numbers of unique OTUs in the RGS and FGS (172 and 85, respectively) were higher than those in the other two stages. Besides, we also compared species diversities from different growth stages. In the SS, *Microidium phyllanthi, Pseudopithomyces chartarum, Curvularia senegalensis, Periconia byssoides,* and *Pseudozyma aphidis* were the most abundant species at 600 m, with percentages of 90.43%, 1.92%, 1.22%, 0.38%, and 0.24%, respectively. Similarly, *Microidium phyllanthi, Pseudopithomyces chartarum, Pseudozyma aphidis, Candida albicans*,and *Penicillium oxalicum* were the top five most abundant species at 1000 m, representing 94.80%, 0.36%, 0.13%, 0.07%, and 0.07% of the total species, respectively. At 1300 m, *Microidium phyllanthi* (90.97%) was the most abundant species, followed by *Cladosporium funiculosum* (1.39%), *Lysurus cruciatus* (0.77%), *Cladosporium allicinum* (0.03%), and *Rhodotorula ingeniosa* (0.03%). In the RGS, *Microidium phyllanthi* (69.19%), *Cladosporium allicinum* (0.03%), *Pseudopithomyces chartarum* (0.29%), *Irpex lacteus* (0.23%), and *Cladosporium funiculosum* (0.08%) were the dominant species at 600 m. At 1000 m, *Microidium phyllanthi*, *Pseudopithomyces chartarum*, *Periconia byssoides*, *Curvularia senegalensis* and *Whalleya microplaca* comprised approximately 84.16%, 0.02%, 0.02%, 0.02%, and 0.02%, respectively, of the total species. In the FGS and MS, *Microidium phyllanthi* and *Pseudopithomyces chartarum* were the dominant species at all three altitudes, comprising more than 94% of the total species (Figures S1–S12). The annotation results of OTUs showed that the species of fungi were most abundant in the RGS. Thus, among the four growth stages examined, we identified a higher abundance of OTUs and species diversity at the RGS stage.

### 2.4. Dissimilarity of Fungal Communities at Different Altitudes

**Figure 4:**
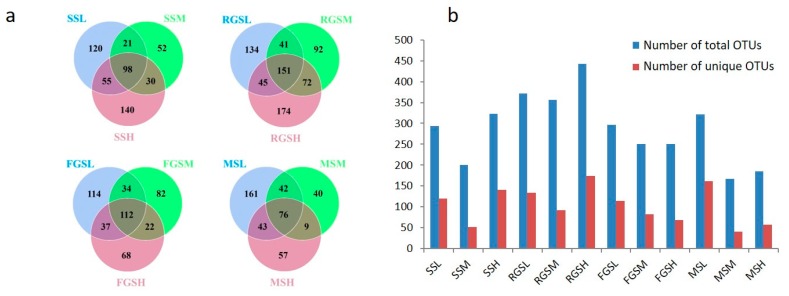
Comparison of fungal communities at different altitudes. (**a**) Venn diagram for samples among different combinations. Each circle represents a sample (group), with the number in the overlapping circles representing the numbers of OTUs shared between those samples (groups), and the number in the non-overlapping regions representing the number of unique OTUs of that sample (group). (**b**) Statistics of the OTUs numbers at different growth stages. Total OTUs refers to all the OTUs in a certain growth stage. Unique OTUs are those specific to a particular growth stage.

In order to explore the effects of different altitudes on the composition of tobacco endophytic fungi, we compared the corresponding number of OTUs and fungal compositions in different altitude locations. The comparison of the OTUs of the different samples at different altitudes indicated that SS samples shared 98 common OTUs, and that samples SSL, SSM, and SSH had 120, 52, and 140 unique OTUs, respectively. For the RGS samples, 151 common OTUs were found at different altitudes. resettling growth stage low (RGSL), resettling growth stage middle (RGSM), and resettling growth stage high (RGSH) samples contained 134, 92, and 174 unique OTUs, respectively. In addition, 112 common OTUs were found among the FGS samples. 114, 82 and 68 OTUs were unique to the fast growth stage low (FGSL), fast growth stage middle (FGSM) and fast growth stage high (FGSH) stages, respectively. Further, 76 OTUs shared in all the samples of MS stages, which were annotated to 45 species (Figure 4a). The variation of the OTUs in SS and RGS decreased between 600 m and 1000 m, then increased at 1300 m. However, the tendency of the OTUs in the FGS and MS samples was decreasing with the increasing growth altitudes (Figure 4b). The fungal communities showed a similar tendency. Therefore, we have a conclusion that the diversity of fungal communities tends to decrease with increasing altitude based on the OTU richness.

### 2.5. Alpha Diversity

**Figure 5:**
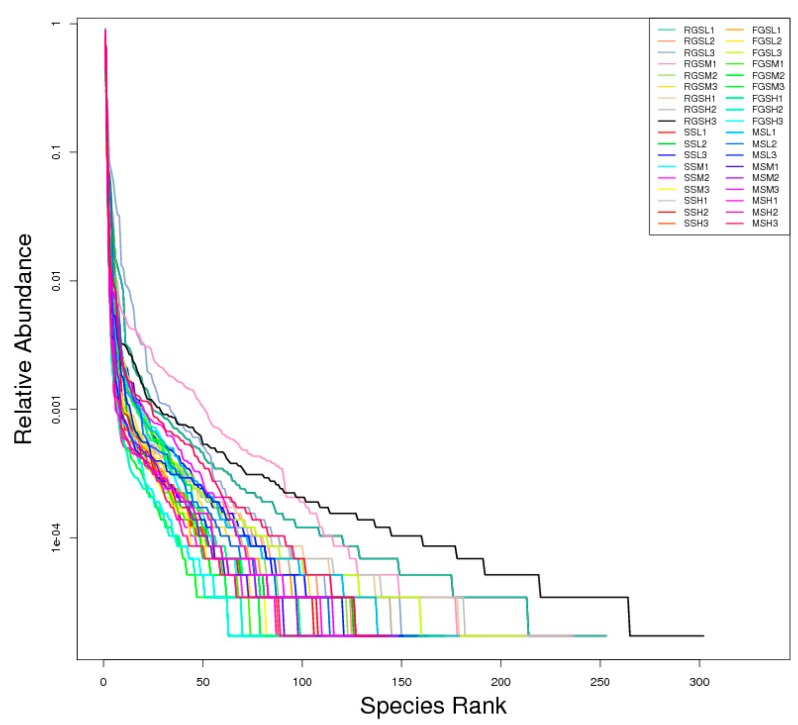
Rank abundance plots of fungal community composition in *Nicotiana tabacum*.

QIIME was used to compare the alpha diversity of all the samples [18]. Rank abundance curves can reflect the richness and evenness of species in the samples (Figure 5). Additionally, the OTU richness was determined by calculating the Observed Species, Shannon, Simpson, Chao1, abundance-based coverage estimator (ACE), Good’s coverage, and PD_whole_tree indices based on the total number of species. The alpha diversity indices are shown in Table 1. The number of Observed Species was highest in the RGSH samples at 238.33 ± 61.61 but was lowest in the MSM samples at 88.67 ± 12.66. Our results showed that RGSL samples had the highest Shannon index values with an average value of 2.21 ± 1.14. In contrast, the lowest Shannon index values (0.90 ± 0.27) were obtained for the MSH samples. We found that RGSH (261.76 ± 59.95) and MSM (99.51 ± 17.63) samples had the highest and lowest Chao1 values, respectively. Similarly, values for the ACE and PD_whole_tree indices were highest for RGSH samples and lowest for MSM samples, with the values of 268.72 ± 60.71/105.90 ± 23.83 and 65.16 ± 8.26/31.27 ± 5.26, respectively. The Simpson indices of all the samples ranged from 0.25 to 0.52, whereas we found little variation in the values of Good’s coverage of all samples, all of which were nearly equal to one. Our goal was to compare fungal communities at different growth stages and examine the effects of altitude on fungal communities in tobacco leaves. Therefore, the variation in all the indices of alpha diversity was compared. The results showed that the indices were highest for the RGS samples, followed by the MS samples. For example, average values for observed species in SS, RGS, FGS, and MS were 143.89 ± 17.20, 212.89 ± 50.95, 141.49 ± 17.46, and 118.56 ± 24.50, respectively (Table S3). Statistical analysis of the alpha diversity indices showed that RGS samples had significantly different numbers of species compared with samples from other stages (*p* < 0.05) (Table S4). These trends were consistent with the change in abundance of the OTUs. At the same time, variation in the alpha diversity indices at different altitudes in SS, FGS, and MS samples was observed; nearly all the alpha diversity indices indicated that the diversity of fungal communities decreased with increasing altitude in these three growth stages (Table 1).

### 2.6. Beta Diversity

**Figure 6:**
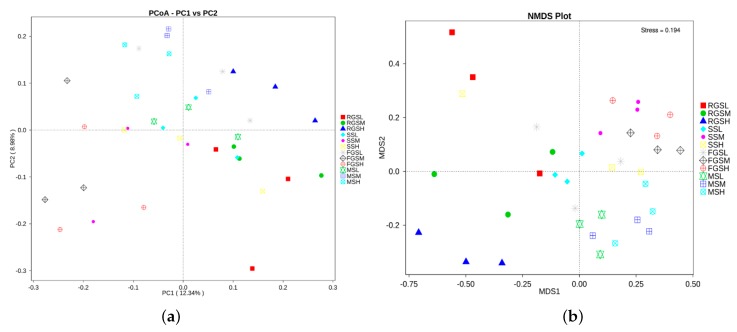
The beta diversity indices of different samples of *Nicotiana tabacum* (**a**) Principal coordinate analysis (PCoA) based on the weighted and unweighted Unifrac distances. (**b**) NMDS analysis results based on operational taxonomic unit (OTU) levels.

**Figure 7:**
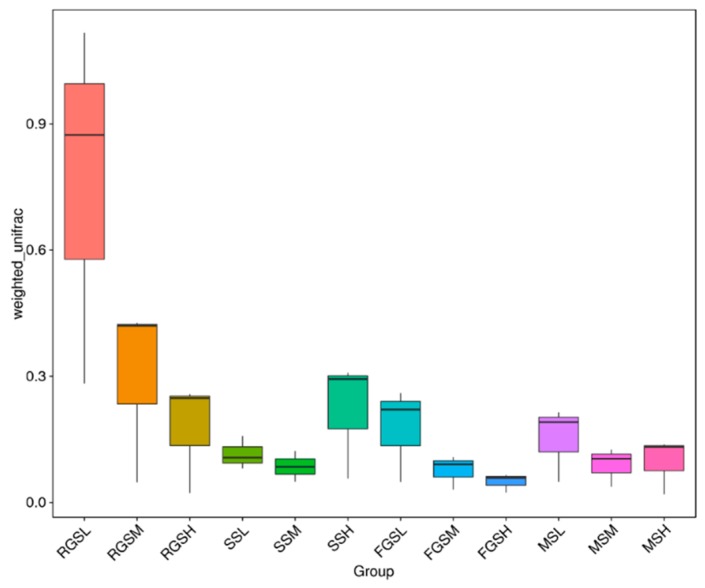
Boxplots of beta diversity based on the weighted Unifrac distances.

To determine beta diversity, we initially obtained information on species abundance based on the annotation results of all the species and the abundance information of OTUs (Table S2). Meanwhile, the other two important indices of beta diversity-Unifrac distance and Distance matrices were also calculated in this study [19,20]. Finally, principal coordinates analysis (PCoA) and nonmetric multidimensional scaling (NMDS) were used to show the differences between different samples. The results of the PCoA based on unweighted Unifrac distances demonstrated that MSL, MSM, and MSH samples tended to cluster together. A similar pattern was observed in the other three stages (Figure 6). The highest variations in the microbiota of different samples were 12.34% (PC1) and 8.98% (PC2), representing a strong separation based on the different stages and altitudes (Figure 6a). NMDS is a ranking method that is applicable to ecology. Nonlinear NMDS models can overcome the shortcomings of linear models and reflect the nonlinear structure of ecological data [21]. NMDS analysis results based on OTU levels were shown in Figure 6b. These results indicated that different growth stages play an important role in shaping fungal communities in *N. tabacum*. The stress of all samples in our study was 0.194, which can accurately reflect the differences among the samples. The results showed that there were high similarities among the samples collected at different growth stages. On the basis of the similarities of fungal communities, samples were divided into three groups, namely, samples RGSL, RGSM, and RGSH; and the remaining samples formed a community. These clusters confirmed that growth stage is the most important factor influencing the elevation in beta diversity among the fungal communities of the multiple samples. Both the results of PCoA and NMDS showed that the higher the elevations are, the smaller the differences between samples. In order to clearly compare the beta diversity among samples, we generated boxplots to show the variation among all samples. The weighted UniFrac distance indicated that the RGSL sample had the highest variation (Figure 7). Furthermore, we identified a trend indicating that the diversity of fungal communities tended to decrease with increasing tobacco growth altitude. Interestingly, the fungal communities in the samples were highly similar, apart from samples FGSH and RGSL (*p* = 0.0053), FGSM and RGSL (*p* = 0.0151), MSH and RGSL (*p* = 0.0285), MSM and RGSL (*p* = 0.0218), and RGSL and SSM (*p* = 0.0239) (Table S5), which suggests that the fungal communities in these samples are differentiated from each other. Overall, there was a higher abundance of fungal species in the RGS samples and a clear trend of decreasing fungal community diversity with increasing altitude, based on the Unifrac distances.

## 3. Discussion

Currently, it has been demonstrated that endophytic fungi play important roles in providing nutrients to their hosts, adapting hosts to their environments, defending hosts from biotic and abiotic stresses, and promoting plant community biodiversity [22], but with limited research focusing on the diverse communities and indispensable functions in tobacco, even though some studies demonstrated that metabolites of tobacco endophytic fungi may play an important role in the growth and biological prevention and control [23]. However, there still lacks a comprehensive study on the fungal communities in tobacco. Therefore, we performed a comprehensive comparison of the fungal communities of tobacco at four growth stages and three altitudes. For the study on the endophytic fungi in tobacco, Pei et al. isolated and analyzed the dynamic population of endophytic fungi in different organs of tobacco and found that the abundance of endophytic fungi in tobacco leaves were the highest and *Alternaria* and *Chaetomium* were the dominant genera [24]. In contrast, our study showed that *Microidium*, *Cladosporium*, *Pseudopithomyces*, *Irpex*, and *Lysurus* were the top five genera according to their abundances using a high-throughput sequencing technique. This difference may be a result of different methods having different detection capabilities for endophytic fungi. The results of our study imply that these top genera may play critical roles in maintaining the structure and function of fungal communities in tobacco. So, what roles do these dominant flora play in tobacco? Within these genera, *Irpex lacteus* is a robust fungus that is efficient at degrading various toxic compounds, colonizing soil, inhibiting soil bacteria, etc. These characteristics make it suitable for water and soil bioremediation for organisms [25,26]. Combined with the existing functions, we speculated that *I. lacteus* may play a similar role in the leaves of tobacco, such as removing the effects of polycyclic aromatic hydrocarbons on the leaves by exerting the activity of manganese peroxidase. Unfortunately, the functions of the other four genera are still unknown and need further exploration. In addition, we also found some endophytic fungi that possess potential ability in resisting abiotic and abiotic stress in tobacco. Previous studies demonstrated that the fermentation products of the endophytic fungus *Aspergillus* can inhibit the tobacco mosaic virus [27]. Besides, it has been demonstrated that the contents of aldehydes and ketones in the tobacco were doubled by the solid fermentation product from *Aspergillus* [28]. A total of 10 species belonging to *Aspergillus* were found in our study. We speculated that their secondary metabolites may play a similar role in tobacco. Moreover, three species of *Fusarium* were detected, which has been shown to reduce the heavy metal content of plants [29]. A large number of endophytic fungi have been identified in tobacco, but further research and clarification are needed to elucidate their roles and functions in tobacco.

Moreover, our results showed that the RGS stage possessed the highest abundance of fungal species. The high richness at the RGS stage may be due to the fact that fungal growth rate will be accelerated after tobacco is transplanted into fields, at which time plant vegetative growth predominates, and there is a high requirement for water and nutrients. Therefore, a diverse group of fungal species can readily form at the RGS stage compared with other stages. Besides, after the seedlings have been transplanted and the fungicidal treatment discontinued, endophytic fungi can colonize in the leaves [3]. In contrast, the lowest abundance of endophytes was detected at the MS stage. In addition, we found higher numbers of unique fungi at the RGS and FGS stages compared with the other two stages. Given that these two stages coincide with a period of rapid plant growth, endophytic fungi may thus be associated with the promotion of growth in tobacco, which would be consistent with the findings of a previous study [23]. The general trend observed in the variation of endophytes in tobacco is that the young tissues or fast growth stages tend to have higher fungal diversity than the mature tissues. By the time of MS, the whole plant has changed from vegetative growth to reproductive growth, and the overall growth trend also stabilized. Correspondingly, the endophytic flora closely related to it also tends to be stable. This phenomenon is further supported by the findings of Houlden et al., who detected a decrease in the diversity of symbiotic fungal species with plant maturity, which has also been found in mycorrhizal associations as well as rhizosphere communities [30]. Our results also showed clear differences in the relative abundances of certain species among the four growth stages examined, suggesting that some endophyte species may preferentially proliferate at a certain stage and play ecological roles that are distinct from those of other endophytes.

At the SS and RGS, the diversity of endophytic fungi showed an initial decrease and then increased with an increase in altitude. In contrast, at the FGS and MS stages, the diversity of endophytic fungi in tobacco showed a decreasing trend with increasing altitude, which was paralleled by a decrease trend in alpha diversity. Overall, we identified a trend whereby the diversity of fungal communities tended to decrease with an increase in the altitude at which tobacco was grown. This observation is consistent with the findings of previous studies that altitude can influence the ecosystem by regulating the living environment through climatic factors and soil formation [31,32]. Similarly, previous studies have demonstrated that the physical and chemical properties of soil change with an increase of altitude, and that this is associated with a decrease in microbial community abundance and diversity [33,34]. Our results showed that there were significant differences in the functional diversity of fungal communities at different elevations.

## 4. Materials and Methods

### 4.1. Material Collection

A total of 36 tobacco leaves were collected from April to August in 2016 from the Modern Tobacco Agricultural Science and Technology Demonstration Garden on Wangcheng Slope, Enshi, Hubei (108°23′12″–110°38′08″ E, 29°07′10″–31°24′13″ N). These 36 samples were collected from four growth stages, the seedling stage (SS), resettling growth stage (RGS), fast-growing stage (FGS), and maturing stage (MS), grown at three altitudes (600, 1000, and 1300 m). To ensure that the experiment was representative, we randomly selected three plants at each geographic location for each growth phase. For example, we firstly selected three strains randomly in this position when we collected the tobacco leaves of the seedling stages at 600 m. Then, we collected upper, middle, and lower leaves equivalent from one strain and then mixed them for a total weight of 100 mg for the following experiments. The materials from the other growth stages and altitudes were also collected as described above. Meanwhile, in order to remove other microbial interference on the surface of tobacco leaves (epiphytic fungi), the surface sterilization procedure with 70% EtOH for 15 s was conducted. After this, the tobacco leaves were washed with filtered water three times and then wiped off with filter paper before DNA extraction. All samples were immediately put on ice and then stored at −80 °C as soon as possible until total DNA extraction. All samples representing different growth stages and altitudes were collected in three replicates. The samples from the seedling stage at 600 m (L), 1000 m (M), and 1300 m (H) were designated as SSL1, SSL2, SSL3, SSM1, SSM2, SSM3, SSH1, SSH2, SSH3. Similarly, samples collected from the other three growth stages were designated as RGSL1, RGSL2, RGSL3, RGSM1, RGSM2, RGSM3, RGSH1, RGSH2, and RGSH3; FGSL1, FGSL2, FGSL3, FGSM1, FGSM2, FGSM3, FGSH1, FGSH2, and FGSH3; and MSL1, MSL2, MSL3, MSM1, MSM2, MSM3, MSH1, MSH2, and MSH3.

### 4.2. DNA Extraction and PCR Amplification

Approximately 100 mg of tobacco leaves were used for each individual DNA extraction. DNA was extracted using the cetyltrimethylammonium bromide (CTAB)method [35]. After extraction, the integrities of DNA were determined using 1% agarose gel electrophoresis. The purity and concentration of DNA were determined using a NanoDrop 2000c spectrophotometer. Each DNA sample was diluted to a final concentRAtion of 1 ng/μL with sterile distilled water and then was used as a DNA template. ITS1 regions of endophytic fungi sequencing were generated by the PCR primers pair CTTGGTCATTTAGAGGAAGTAA and GCTGCGTTCTTCATCGATGC. This amplification was conducted using these primers without the sequencing adapters and sample-specific barcodes. The PCR reactions were carRIed out in 20 μL reaction mixtures, COntaining 5.8 μL sterile distillED water, 10.0 μL 5× Phusion^®^ High-Fidelity PCR Master Mix with GC Buffer (New England Biolabs, Ipswich, Ma, USA), 1.0 μL forwARd primeR (10 μM), 1.0 μL reveRSe primeR (10 μM), 0.2 μL Taq DNA PolymERase, and 2.0 μL template DNA. The PCR amplification conditions were as follows: initial denaturation at 95 °C for 2 min, 30 cycles of denaturation at 95 °C for 30 s, annealing at 50 °C for 20 s, elongation at 72 °C for 60 s, elongation at 72 °C for 10 min, and then held at 4 °C.

### 4.3. Library Construction and Sequencing

Following the above amplification, the PCR amplicons were detected using 2% agarose gel electrophoresis. Subsequently, adapters including sample-specific barcodes were added to these amplicons by PCR amplification. PCR cycling conditions were the same as described above, except that the number of PCR cycles was changed to 20. After that, all the amplicons were then pooled in equimolar ratios into a single tube. The mixed amplicons were further detected using 2% agarose gel electrophoresis and the target sequences were extracted using a QIAquick Gel Extraction Kit (Qiagen, 28704, Munich, Germany). The libraries were constructed using a TruSeq^®^ DNA PCR-Free Sample Preparation Kit (Illumina, 20015963, San Diego, CA, USA). The libraries were quantified using a Qubit^®^ 2.0 Fluorometer (Life Technologies, Carlsbad, CA, USA) and qPCR, and then sequenced using the Illumina HiSeq 2500 platform. All sequence data have been submitted to the Sequence Read Archive (SRA: SRP145216) and are freely available at the NCBI (BioProject: PRJNA464258).

### 4.4. Data Processing

To perform an accurate taxonomic assignment for each sequence, quality control and length trimming for the raw reads were needed. Briefly, the qualities of raw reads were trimmed by removing reads shorter than 200 bases and reads containing ambiguous bases. Meanwhile, primers and barcodes were removed. Sequences that passed the pre-processing were used for the following analyses. The remaining reads of each sample were then assembled using FLASH (V1.2.7) (http://ccb.jhu.edu/software/FLASH/) [36] to generate raw tags. QIIME (V1.7.0) (http://qiime.org/scripts/split_libraries_fastq.html) [19] was used with the following parameters for quality filtering of the tags: (a) tag truncating: raw tags from the continuous low-quality value (the default quality value ≤ 19) with the setting length (the default value is 3) were truncated at the first low-quality base site; (b) tag length filtering: removing the length <75% tags. Chimeras were checked against the Unite database (https://unite.ut.ee/) [37] using the UCHIME Algorithm (http://www.drive5.com/usearch/manual/uchime_algo.html) [38] and then removed. Finally, the effective tags were obtained.

### 4.5. OTU Clustering and Annotation

Uparse (Uparse v7.0.1001, http://drive5.com/uparse/) [39] was used to cluster all the effective tags. The effective tags with 97% identity were clustered into the same OTUs. The OTUs with the highest frequency were selected as the representative of OTU sequences. We removed OTUs with only one sequence from the dataset since these unique OTUs could result from sequencing errors. For a further determination of their function, these OTUs were annotated using the blast method with QIIME (Version 1.7.0) [19,20,21] based on the Unite database (https://unite.ut.ee/) [40]. Thereafter, the fungal communities at different taxonomic levels (kingdom, phylum, class, order, family, genus, and species) were counted. The phylogenetic relationships among all the OTUs were initially blasted using MUSCLE (Version 3.8.31, http://www.drive5.com/muscle/) [41], and then we analyzed their phylogenetic relationships using MrBayes 3 [42]. For the comparison of the OTUs and fungal communities at different altitudes and growth stages, we used Venn diagrams (http://bioinformatics.psb.ugent.be/webtools/Venn/).

### 4.6. Alpha Diversity

The Observed Species, Chao-1, Shannon, Simpson, ACE, Good’s -coverage, and PD_whole_tree diversity indices were determined using QIIME (Version 1.7.0) [19]. Chao and ACE indices were used to determine community richness, and Shannon and Simpson indices were used to determine community diversity. These metrics are useful for estimating microbial diversity and richness. Coverage represents the sequencing depth. The PD_whole_tree index was used to compute Faith’s phylogenetic diversity metric. R software (Version 2.15.3, R Foundation for Statistical Computing, Vienna, Austria) was used to draw the dilution, rank abundance, and species accumulation curves, and to analyze the difference in alpha diversity indices among all groups.

### 4.7. Beta Diversity

Beta diversity is a comparative analysis of microbial community constitutions for all different samples. To determine beta diversity, we initially obtained information on species abundance based on the annotation results of all the species and the abundant information of OTUs. Unifrac distance refers to the relative relatedness of OTUs in a community and was calculated from the sample sequences. Unifrac distance metrics were used in an Unweighted Pair Group Method with Arithmetic Mean (UPGMA) using QIIME software (Version 1.7.0) [19]. PCoA and NMDS were used to show the differences between different samples. Subsequently, the ade4 and ggplot2 packages, WGCNA, stats and ggplot2 packages, and vegan packages of R software (Version 2.15.3) were used to draw principal coordinate analysis (PCoA) and NMDS diagrams, respectively. In order to obtain a better insight into the clustering of fungal communities, both weighted (takes into account changes in relative taxon abundance) and unweighted UniFrac metrics were used for beta diversity [20]. Metastats analysis was performed on different taxonomic levels (phylum, class, order, family, genus, and species), using the permutation test between groups. Statistical analyses were performed in R 2.15.1 (The R Foundation for Statistical Computing, Vienna, Austria).

## 5. Conclusions

In this study, we analyzed and quantified the endophytic fungi of healthy tobacco leaves from SS, RGS, FGS, and MS at three altitudes (600, 1000, and 1300 m) using a high-throughput sequencing technique. Our results indicated that tobacco is host to an abundant community of endophytic fungi, which provides new opportunities for exploring their potential utilization. In future studies, more tissues and environmental factors should be integrated to better characterize the distributions and functions of the endophytic fungal community in tobacco.

## Figures and Tables

**Table 1:** Median alpha diversity indices.

Sample Name	Observed Species	Shannon	Simpson	Chao1	ACE	Good’s Coverage	PD_Whole Tree
SSL	161.33 ± 11.59	1.24 ± 0.23	0.31 ± 0.06	188.74 ± 6.69	202.87 ± 18.15	0.99 ± 0	48.24 ± 2.25
SSM	108.67 ± 15.95	0.98 ± 0.36	0.26 ± 0.10	120.22 ± 22.99	126.28 ± 27.77	0.99 ± 0	39.10 ± 6.89
SSH	161.67 ± 24.08	1.15 ± 0.54	0.29 ± 0.14	183.57 ± 23.35	193.26 ± 18.65	0.99 ± 0	49.96 ± 9.17
RGSL	204.54 ± 69.00	2.21 ± 1.14	0.52 ± 0.28	232.33 ± 65.59	246.19 ± 76.29	0.99 ± 0	56.10 ± 11.99
RGSM	196.33 ± 36.56	1.75 ± 0.88	0.40 ± 0.17	231.17 ± 54.97	247.21 ± 51.28	0.99 ± 0	52.26± 4.58
RGSH	238.33 ± 61.61	1.28 ± 0.60	0.29 ± 0.12	261.76 ± 59.95	268.72 ± 60.71	0.99 ± 0	65.16 ± 8.26
FGSL	162.67 ± 14.57	1.29 ± 0.30	0.33 ± 0.09	196.60 ± 28.75	198.75 ± 18.52	0.99 ± 0	50.46 ± 5.90
FGSM	134.13 ± 18.52	1.08 ± 0.34	0.29 ± 0.09	159.54 ± 29.82	169.47 ± 30.37	0.99 ± 0	51.84 ± 3.10
FGSH	127.67 ± 19.30	1.05 ± 0.40	0.28 ± 0.09	136.94 ± 21.49	141.81 ± 20.66	1.00 ± 0	48.14 ± 6.40
MSL	164.67 ± 44.77	1.20 ± 0.42	0.30 ± 0.12	188.69 ± 60.70	196.37 ± 57.17	0.99 ± 0	54.48 ± 7.73
MSM	88.67 ± 12.66	0.94 ± 0.27	0.25 ± 0.06	99.51 ± 17.63	105.90 ± 23.83	1.00 ± 0	31.27 ± 5.26
MSH	102.33 ± 26.08	0.90 ± 0.27	0.25 ± 0.07	122.94 ± 29.65	127.68 ± 34.26	0.99 ± 0	35.47 ± 6.91

All data presented in text and tables are expressed as means ± standard deviation (SD).

## Supplementary Materials

Supplementary materials can be found at http://www.mdpi.com/1422-0067/19/11/3421/s1.
