# Supplementary material for: Composition and Genetic Diversity of the Nicotiana tabacum Microbiome in Different Topographic Areas and Growth Periods
Source: Int J Mol Sci. 2018 Oct 31;19(11):3421. doi: 10.3390/ijms19113421 (PMC6275082; doi:10.3390/ijms19113421)
Supplement: Supplementary file 1 [file ijms-19-03421-s001.zip › ijms-348151-supplementary-final check/Supporting imformation-20181026/Table S1 Statistics of the sequencing results.docx]

| Sample Name | Raw PE | Combined | Qualified | Nochime | Base | AvgLen | Q20 | Q30 | GC% | Effective  % |
| --- | --- | --- | --- | --- | --- | --- | --- | --- | --- | --- |
| FGSH1 | 70,606 | 68,744 | 65,512 | 65,462 | 20,778,966 | 317 | 99.06 | 97.89 | 60.06 | 92.71 |
| FGSH2 | 80,816 | 79,179 | 76,757 | 76,748 | 24,430,619 | 318 | 99.05 | 97.85 | 60.53 | 94.97 |
| FGSH3 | 71,931 | 70,417 | 68,369 | 68,349 | 21,731,260 | 318 | 99.09 | 97.95 | 60.45 | 95.02 |
| FGSL1 | 74,679 | 71,881 | 68,222 | 68,180 | 21,593,884 | 317 | 99.01 | 97.82 | 60.14 | 91.3 |
| FGSL2 | 88,366 | 85,086 | 81,407 | 81,339 | 25,804,619 | 317 | 99.03 | 97.85 | 60.26 | 92.05 |
| FGSL3 | 86,122 | 74,890 | 65,367 | 65,075 | 20,228,233 | 311 | 98.99 | 97.79 | 58.9 | 75.56 |
| FGSM1 | 75,716 | 73,401 | 70,518 | 70,501 | 22,384,915 | 318 | 98.89 | 97.55 | 60.34 | 93.11 |
| FGSM2 | 80,383 | 77,635 | 75,230 | 75,155 | 23,861,206 | 317 | 99.05 | 97.85 | 60.42 | 93.5 |
| FGSM3 | 76,072 | 73,582 | 69,611 | 69,568 | 21,998,613 | 316 | 99.06 | 97.89 | 59.86 | 91.45 |
| MSH1 | 86,468 | 84,562 | 82,038 | 82,010 | 26,090,310 | 318 | 99 | 97.76 | 60.44 | 94.84 |
| MSH2 | 70,926 | 69,152 | 67,290 | 67,241 | 21,388,961 | 318 | 99.08 | 97.91 | 60.47 | 94.8 |
| MSH3 | 70,787 | 68,469 | 65,262 | 65,199 | 20,580,785 | 316 | 99.05 | 97.88 | 59.7 | 92.11 |
| MSL1 | 72,730 | 69,969 | 65,768 | 65,646 | 20,792,604 | 317 | 98.94 | 97.68 | 60.02 | 90.26 |
| MSL2 | 87,026 | 84,328 | 80,140 | 80,076 | 25,229,945 | 315 | 99.01 | 97.85 | 59.44 | 92.01 |
| MSL3 | 89,936 | 87,482 | 84,215 | 84,146 | 26,722,452 | 318 | 98.93 | 97.66 | 60.24 | 93.56 |
| MSM1 | 73,651 | 72,155 | 70,017 | 69,936 | 22,239,541 | 318 | 99.04 | 97.85 | 60.46 | 94.96 |
| MSM2 | 89,979 | 87,466 | 84,799 | 84,777 | 26,945,697 | 318 | 99.01 | 97.83 | 60.31 | 94.22 |
| MSM3 | 78,277 | 75,362 | 72,077 | 72,031 | 22,753,664 | 316 | 99.07 | 97.92 | 59.8 | 92.02 |
| RGSH1 | 87,471 | 84,410 | 81,262 | 81,166 | 25,747,992 | 317 | 99.05 | 97.89 | 60.27 | 92.79 |
| RGSH2 | 72,509 | 69,551 | 67,038 | 66,917 | 21,226,014 | 317 | 98.99 | 97.78 | 60.27 | 92.29 |
| RGSH3 | 71,598 | 64,827 | 58,332 | 58,005 | 18,072,667 | 312 | 98.96 | 97.73 | 58.71 | 81.01 |
| RGSL1 | 78,724 | 75,026 | 73,754 | 73,455 | 22,724,901 | 309 | 98.88 | 97.58 | 58.73 | 93.31 |
| RGSL2 | 76,954 | 74,563 | 70,829 | 70,753 | 22,405,208 | 317 | 98.96 | 97.71 | 60.1 | 91.94 |
| RGSL3 | 89,412 | 80,891 | 79,658 | 79,255 | 22,442,567 | 283 | 99 | 97.92 | 52.88 | 88.64 |
| RGSM1 | 78,016 | 71,058 | 63,055 | 62,549 | 19,193,123 | 307 | 98.93 | 97.74 | 57.13 | 80.17 |
| RGSM2 | 84,338 | 81,287 | 77,909 | 77,843 | 24,580,960 | 316 | 98.98 | 97.76 | 59.71 | 92.3 |
| RGSM3 | 87,971 | 84,473 | 80,115 | 79,809 | 25,106,288 | 315 | 98.97 | 97.75 | 59.59 | 90.72 |
| SSH1 | 79,319 | 73,266 | 72,192 | 72,035 | 21,811,438 | 303 | 98.99 | 97.79 | 58.49 | 90.82 |
| SSH2 | 87,987 | 84,637 | 79,379 | 79,335 | 25,122,046 | 317 | 98.98 | 97.73 | 60.22 | 90.17 |
| SSH3 | 86,889 | 84,421 | 81,442 | 81,413 | 25,873,384 | 318 | 98.98 | 97.72 | 60.43 | 93.7 |
| SSL1 | 86,288 | 82,900 | 79,785 | 79,734 | 25,211,232 | 316 | 98.94 | 97.68 | 60.05 | 92.4 |
| SSL2 | 79,819 | 75,080 | 71,918 | 71,639 | 22,374,035 | 312 | 98.99 | 97.77 | 59.3 | 89.75 |
| SSL3 | 84,526 | 80,186 | 75,325 | 75,231 | 23,620,753 | 314 | 99 | 97.78 | 59.67 | 89 |
| SSM1 | 87,391 | 83,740 | 79,585 | 79,512 | 25,060,723 | 315 | 99.01 | 97.81 | 59.69 | 90.98 |
| SSM2 | 71,521 | 69,064 | 65,837 | 65,742 | 20,875,399 | 318 | 98.94 | 97.65 | 60.46 | 91.92 |
| SSM3 | 73,280 | 70,687 | 67,628 | 67,599 | 21,344,889 | 316 | 99 | 97.79 | 60.12 | 92.25 |

Table S1 Statistics of the sequencing results
