# Supplementary material for: Composition and Genetic Diversity of the Nicotiana tabacum Microbiome in Different Topographic Areas and Growth Periods
Source: Int J Mol Sci. 2018 Oct 31;19(11):3421. doi: 10.3390/ijms19113421 (PMC6275082; doi:10.3390/ijms19113421)
Supplement: Supplementary file 1 [file ijms-19-03421-s001.zip › ijms-348151-supplementary-final check/Supporting imformation-20181026/Table S2 Annotation Results of OTUs.docx]

| Taxonomy | Microidium phyllanthi | Cladosporium allicinum | Pseudopithomyces chartarum | Irpex lacteus | Cladosporium funiculosum | Lysurus cruciatus | Curvularia senegalensis | Rhodotorula ingeniosa | Periconia byssoides | Paraphaeosphaeria michotii | Others |
| --- | --- | --- | --- | --- | --- | --- | --- | --- | --- | --- | --- |
| RGSL | 0.691914 | 0.032633 | 0.029989 | 0.023709 | 0.008193 | 0 | 0.001976 | 0.000893 | 0.00208 | 0.003226 | 0.205387 |
| RGSM | 0.841587 | 0 | 0.002241 | 0.001567 | 0.000012 | 0.000282 | 0.002097 | 0.000006 | 0.002235 | 0.000115 | 0.149857 |
| RGSH | 0.918883 | 0 | 0.001861 | 0.001227 | 0.000006 | 0 | 0.001567 | 0 | 0.001492 | 0.000138 | 0.074825 |
| SSL | 0.904255 | 0 | 0.019249 | 0.000202 | 0.000012 | 0.000012 | 0.01218 | 0 | 0.003774 | 0.000098 | 0.06022 |
| SSM | 0.947996 | 0 | 0.003647 | 0.000398 | 0 | 0.000104 | 0.000069 | 0 | 0.000444 | 0.000058 | 0.047285 |
| SSH | 0.909717 | 0.003284 | 0.001262 | 0.000035 | 0.013943 | 0.007749 | 0.000006 | 0.003013 | 0.00087 | 0.000081 | 0.060041 |
| FGSL | 0.911457 | 0.000012 | 0.038153 | 0.000138 | 0 | 0.000225 | 0.000086 | 0 | 0.000818 | 0.000484 | 0.048627 |
| FGSM | 0.957192 | 0 | 0.011719 | 0 | 0.000006 | 0.000121 | 0.001026 | 0.000006 | 0.000651 | 0.000006 | 0.029274 |
| FGSH | 0.972564 | 0 | 0.002633 | 0.000086 | 0 | 0.000173 | 0.000023 | 0 | 0.000611 | 0.000115 | 0.023795 |
| MSL | 0.913174 | 0 | 0.027805 | 0.000052 | 0.000115 | 0.013551 | 0 | 0.00004 | 0.000761 | 0.00015 | 0.044352 |
| MSM | 0.953216 | 0 | 0.012111 | 0.000374 | 0 | 0.000161 | 0.000035 | 0 | 0.000513 | 0.000035 | 0.033555 |
| MSH | 0.958119 | 0 | 0.007213 | 0.00023 | 0.000006 | 0.000328 | 0.000461 | 0.000006 | 0.003388 | 0.000017 | 0.030231 |

Table S3 Annotation Results of OTUs
