# Supplementary material for: Composition and Genetic Diversity of the Nicotiana tabacum Microbiome in Different Topographic Areas and Growth Periods
Source: Int J Mol Sci. 2018 Oct 31;19(11):3421. doi: 10.3390/ijms19113421 (PMC6275082; doi:10.3390/ijms19113421)
Supplement: Supplementary file 1 [file ijms-19-03421-s001.zip › ijms-348151-supplementary-final check/Supporting imformation-20181026/Table S3 The indices of alpha diversity of different growth stages.docx]

Table S3 The indices of alpha diversity of different growth stages

| Sample name | Observed species | Shannon | Simpson | Chao1 | ACE | Good’s coverage | PD_whole tree |
| --- | --- | --- | --- | --- | --- | --- | --- |
| SS | 143.89±17.20 | 1.12±0.37 | 0.29±0.10 | 164.18±17.68 | 174.14±21.52 | 0.99±0 | 45.77±6.10 |
| RGS | 212.89±50.95 | 1.75±0.87 | 0.40±0.19 | 241.75±60.17 | 254.04±62.76 | 0.99±0 | 57.84±8.27 |
| MS | 141.49±17.46 | 1.14±0.35 | 0.3±0.09 | 164.36±26.69 | 170.01±23.18 | 0.99±0 | 50.15±5.13 |
| FGS | 118.56±24.50 | 1.01±0.32 | 0.27±0.08 | 137.05±32.66 | 143.32±38.42 | 0.99±0 | 40.41±6.63 |
