# Supplementary material for: Composition and Genetic Diversity of the Nicotiana tabacum Microbiome in Different Topographic Areas and Growth Periods
Source: Int J Mol Sci. 2018 Oct 31;19(11):3421. doi: 10.3390/ijms19113421 (PMC6275082; doi:10.3390/ijms19113421)
Supplement: Supplementary file 1 [file ijms-19-03421-s001.zip › ijms-348151-supplementary-final check/Supporting imformation-20181026/Table S4 Statistical analysis of the indices of alpha diversity.docx]

Table S4 Statistical analysis of the indices of alpha diversity at different growth stages and altitudes

Table S4-1 Statistical analysis of the indices of alpha diversity at different altitudes

|  | Observed species _pvalue | sig. | Shannon_pvalue | sig. | Simpson_pvalue | sig. | Chao1_pvalue | sig. | ACE_pvalue | sig. | Good's coverage_pvalue | sig. | PD_whole tree _pvalue | sig. |
| --- | --- | --- | --- | --- | --- | --- | --- | --- | --- | --- | --- | --- | --- | --- |
| SSL SSM | 0.0058 | ** | 0.2685 |  | 0.3459 |  | 0.0061 | ** | 0.0073 | ** | 0.0569 | . | 0.1255 |  |
| SSH SSM | 0.0409 | * | 0.5895 |  | 0.6612 |  | 0.0421 | * | 0.047 | * | 0.3273 |  | 0.0604 | . |
| SSH SSL | 0.3924 |  | 0.5634 |  | 0.6094 |  | 0.3989 |  | 0.4105 |  | 0.3273 |  | 0.7049 |  |
| RGSL RGSM | 0.7986 |  | 0.6995 |  | 0.6612 |  | 0.8082 |  | 0.5952 |  | 0.3273 |  | 0.8709 |  |
| RGSH RGSL | 0.4464 |  | 0.2304 |  | 0.1168 |  | 0.3989 |  | 0.4797 |  | 0.3273 |  | 0.1524 |  |
| RGSH RGSM | 0.6105 |  | 0.4093 |  | 0.2483 |  | 0.5453 |  | 0.8591 |  | 1 |  | 0.1136 |  |
| FGSL FGSM | 0.0022 | ** | 0.3481 |  | 0.445 |  | 0.0053 | ** | 0.0063 | ** | 0.0569 | . | 0.0016 | ** |
| FGSH FGSL | 0.0078 | ** | 0.2766 |  | 0.5968 |  | 0.037 | * | 0.047 | * | 0.3273 |  | 0.0041 | ** |
| FGSH FGSM | 0.6105 |  | 0.8771 |  | 0.8121 |  | 0.3989 |  | 0.3784 |  | 0.3273 |  | 0.7049 |  |
| MSL - MSM | 0.113 |  | 0.3578 |  | 0.5968 |  | 0.171 |  | 0.2431 |  | 0.3273 |  | 0.6267 |  |
| MSH - MSL | 0.0534 | . | 0.2377 |  | 0.4558 |  | 0.019 | * | 0.0283 | * | 0.0062 | ** | 0.7866 |  |
| MSH MSM | 0.7021 |  | 0.7869 |  | 0.8263 |  | 0.2804 |  | 0.2668 |  | 0.0569 | . | 0.4509 |  |

Table S4-2 Statistical analysis of the indices of alpha diversity at different growth stages

|  | Observed species _pvalue | sig. | Shannon_pvalue | sig. | Simpson_pvalue | sig. | Chao1_pvalue | sig. | ACE_pvalue | sig. | Good's coverage_pvalue | sig. | PD_whole tree _pvalue | sig. |
| --- | --- | --- | --- | --- | --- | --- | --- | --- | --- | --- | --- | --- | --- | --- |
| FGSL RGSL | 0.2093 |  | 0.2451 |  | 0.2483 |  | 0.2099 |  | 0.2431 |  | 1 |  | 0.9137 |  |
| FGSL MSL | 0.6787 |  | 0.6028 |  | 0.7012 |  | 0.4687 |  | 0.5952 |  | 0.3273 |  | 0.4196 |  |
| FGSL SSL | 0.7021 |  | 0.7427 |  | 0.798 |  | 0.5453 |  | 0.5167 |  | 0.3273 |  | 0.2402 |  |
| MSL RGSL | 0.3924 |  | 0.513 |  | 0.4344 |  | 0.5859 |  | 0.5167 |  | 0.3273 |  | 0.4835 |  |
| MSL SSL | 0.9745 |  | 0.8468 |  | 0.8981 |  | 0.9034 |  | 0.9057 |  | 1 |  | 0.7049 |  |
| RGSL SSL | 0.3754 |  | 0.3986 |  | 0.3645 |  | 0.5062 |  | 0.5952 |  | 0.3273 |  | 0.2844 |  |
| FGSM MSM | 0.0382 | * | 0.5895 |  | 0.5355 |  | 0.0249 | * | 0.0283 | * | 0.0569 | . | 0.0036 | ** |
| FGSM RGSM | 0 | *** | 0.0915 | . | 0.1426 |  | 0.0001 | *** | 0.0001 | *** | 0.0062 | ** | 0.0031 | ** |
| MSH MSM | 0.7021 |  | 0.7869 |  | 0.8263 |  | 0.2804 |  | 0.2668 |  | 0.0569 | . | 0.4509 |  |
| MSM RGSM | 0.0105 | * | 0.2377 |  | 0.3836 |  | 0.037 | * | 0.0249 | * | 0.3273 |  | 0.9568 |  |
| MSM SSM | 0.1687 |  | 0.6995 |  | 0.5843 |  | 0.0987 | . | 0.1193 |  | 0.3273 |  | 0.0213 | * |
| RGSM SSM | 0.0003 | *** | 0.1223 |  | 0.1622 |  | 0.0006 | *** | 0.0005 | *** | 0.0569 | . | 0.0189 | * |
| FGSH MSH | 0.2093 |  | 0.6712 |  | 0.8692 |  | 0.6714 |  | 0.7674 |  | 0.3273 |  | 0.0483 | * |
| FGSH RGSH | 0 | *** | 0.2934 |  | 0.9271 |  | 0.0002 | *** | 0.0005 | *** | 0.0569 | . | 0.0001 | *** |
| FGSH SSH | 0.0235 | * | 0.3986 |  | 0.7839 |  | 0.0613 | . | 0.0675 | . | 0.3273 |  | 0.0271 | * |
| MSH RGSH | 0.0012 | ** | 0.5253 |  | 0.9417 |  | 0.0006 | *** | 0.0011 | ** | 0.0062 | ** | 0.0213 | * |
| MSH SSH | 0.2701 |  | 0.6712 |  | 0.9126 |  | 0.1382 |  | 0.1193 |  | 0.0569 | . | 0.7866 |  |
