# Supplementary material for: Composition and Genetic Diversity of the Nicotiana tabacum Microbiome in Different Topographic Areas and Growth Periods
Source: Int J Mol Sci. 2018 Oct 31;19(11):3421. doi: 10.3390/ijms19113421 (PMC6275082; doi:10.3390/ijms19113421)
Supplement: Supplementary file 1 [file ijms-19-03421-s001.zip › ijms-348151-supplementary-final check/Supporting imformation-20181026/Table S5 Statistical analysis of the indices of beta diversity.docx]

Table S5 Statistical analysis of the indices of beta diversity

|  | pvalue | sig. |  | pvalue | sig. | pvalue | sig. |  |
| --- | --- | --- | --- | --- | --- | --- | --- | --- |
| FGSH - FGSL | 0.1541 |  | FGSM - MSL | 0.4217 |  | MSL - SSM | 0.5456 |  |
| FGSH - FGSM | 0.657 |  | FGSM - MSM | 0.8715 |  | MSM - RGSH | 0.5193 |  |
| FGSH - MSH | 0.469 |  | FGSM - RGSH | 0.4217 |  | MSM - RGSL | 0.0218 | * |
| FGSH - MSL | 0.2173 |  | FGSM - RGSL | 0.0151 | * | MSM - RGSM | 0.2033 |  |
| FGSH - MSM | 0.5456 |  | FGSM - RGSM | 0.1541 |  | MSM - SSH | 0.2033 |  |
| FGSH - RGSH | 0.2173 |  | FGSM - SSH | 0.1541 |  | MSM - SSL | 0.6282 |  |
| FGSH - RGSL | 0.0053 | ** | FGSM - SSL | 0.5193 |  | MSM - SSM | 0.9677 |  |
| FGSH - RGSM | 0.0667 | . | FGSM - SSM | 0.8398 |  | RGSH - RGSL | 0.0847 | . |
| FGSH - SSH | 0.0667 | . | MSH - MSL | 0.6 |  | RGSH - RGSM | 0.5193 |  |
| FGSH - SSL | 0.2807 |  | MSH - MSM | 0.9034 |  | RGSH - SSH | 0.5193 |  |
| FGSH - SSM | 0.5193 |  | MSH - RGSH | 0.6 |  | RGSH - SSL | 0.8715 |  |
| FGSL - FGSM | 0.317 |  | MSH - RGSL | 0.0285 | * | RGSH - SSM | 0.5456 |  |
| FGSL - MSH | 0.469 |  | MSH - RGSM | 0.2475 |  | RGSL - RGSM | 0.2637 |  |
| FGSL - MSL | 0.8398 |  | MSH - SSH | 0.2475 |  | RGSL - SSH | 0.2637 |  |
| FGSL - MSM | 0.3992 |  | MSH - SSL | 0.7162 |  | RGSL - SSL | 0.0615 | . |
| FGSL - RGSH | 0.8398 |  | MSH - SSM | 0.9355 |  | RGSL - SSM | 0.0239 | * |
| FGSL - RGSL | 0.124 |  | MSL - MSM | 0.5193 |  | RGSM - SSH | 1 |  |
| FGSL - RGSM | 0.657 |  | MSL - RGSH | 1 |  | RGSM - SSL | 0.4217 |  |
| FGSL - SSH | 0.657 |  | MSL - RGSL | 0.0847 | . | RGSM - SSM | 0.2173 |  |
| FGSL - SSL | 0.7162 |  | MSL - RGSM | 0.5193 |  | SSH - SSL | 0.4217 |  |
| FGSL - SSM | 0.4217 |  | MSL - SSH | 0.5193 |  | SSH - SSM | 0.2173 |  |
| FGSM - MSH | 0.7772 |  | MSL - SSL | 0.8715 |  | SSL - SSM | 0.657 |  |
