# Supplementary figures and images for: Composition and Genetic Diversity of the Nicotiana tabacum Microbiome in Different Topographic Areas and Growth Periods
Source: Int J Mol Sci. 2018 Oct 31;19(11):3421. doi: 10.3390/ijms19113421 (PMC6275082; doi:10.3390/ijms19113421)

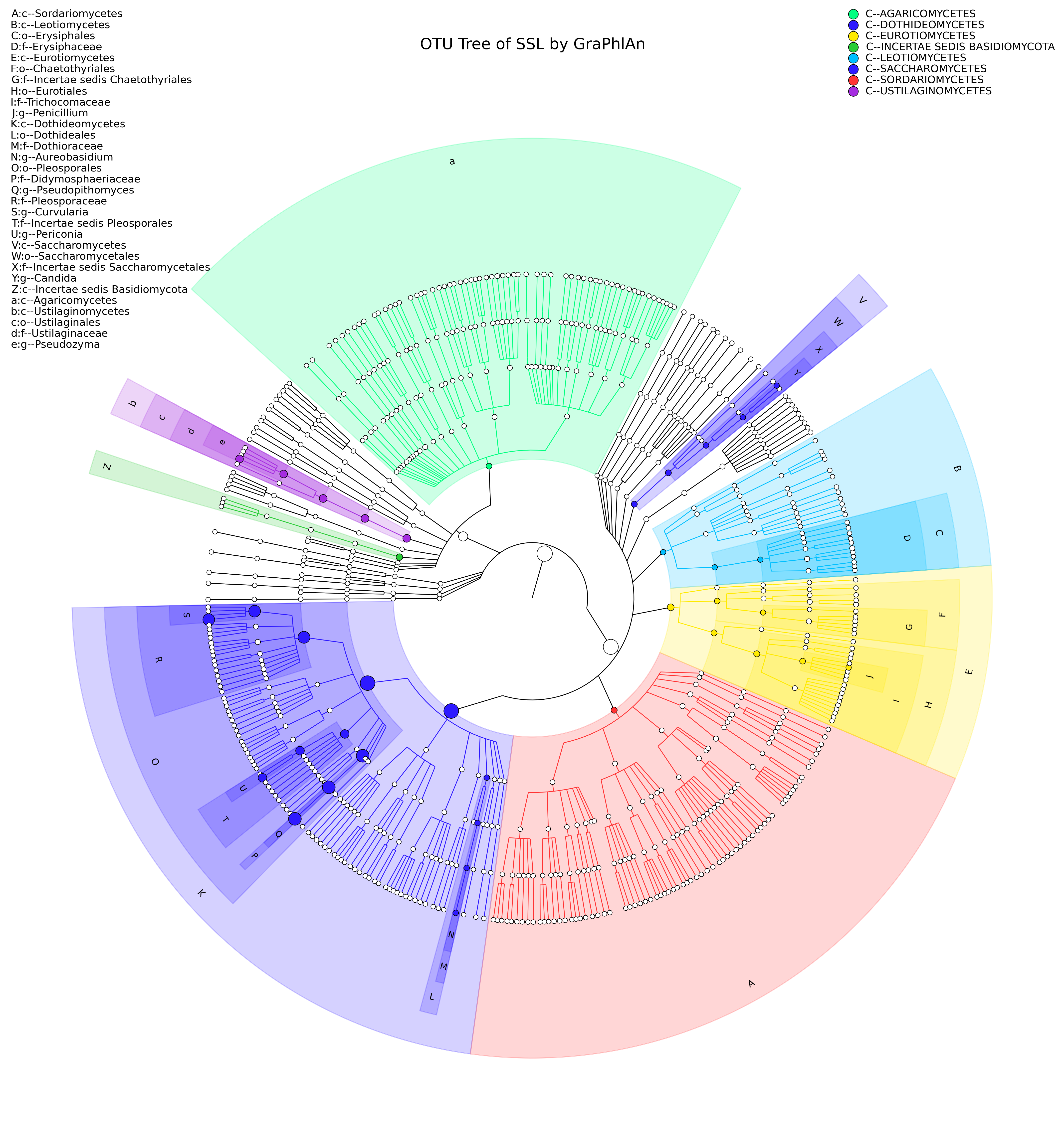

Supplement: Supplementary file 1 [file ijms-19-03421-s001.zip › ijms-348151-supplementary-final check/Supporting imformation-20181026/Figure S1 Hierarchy tree of SSL.png]

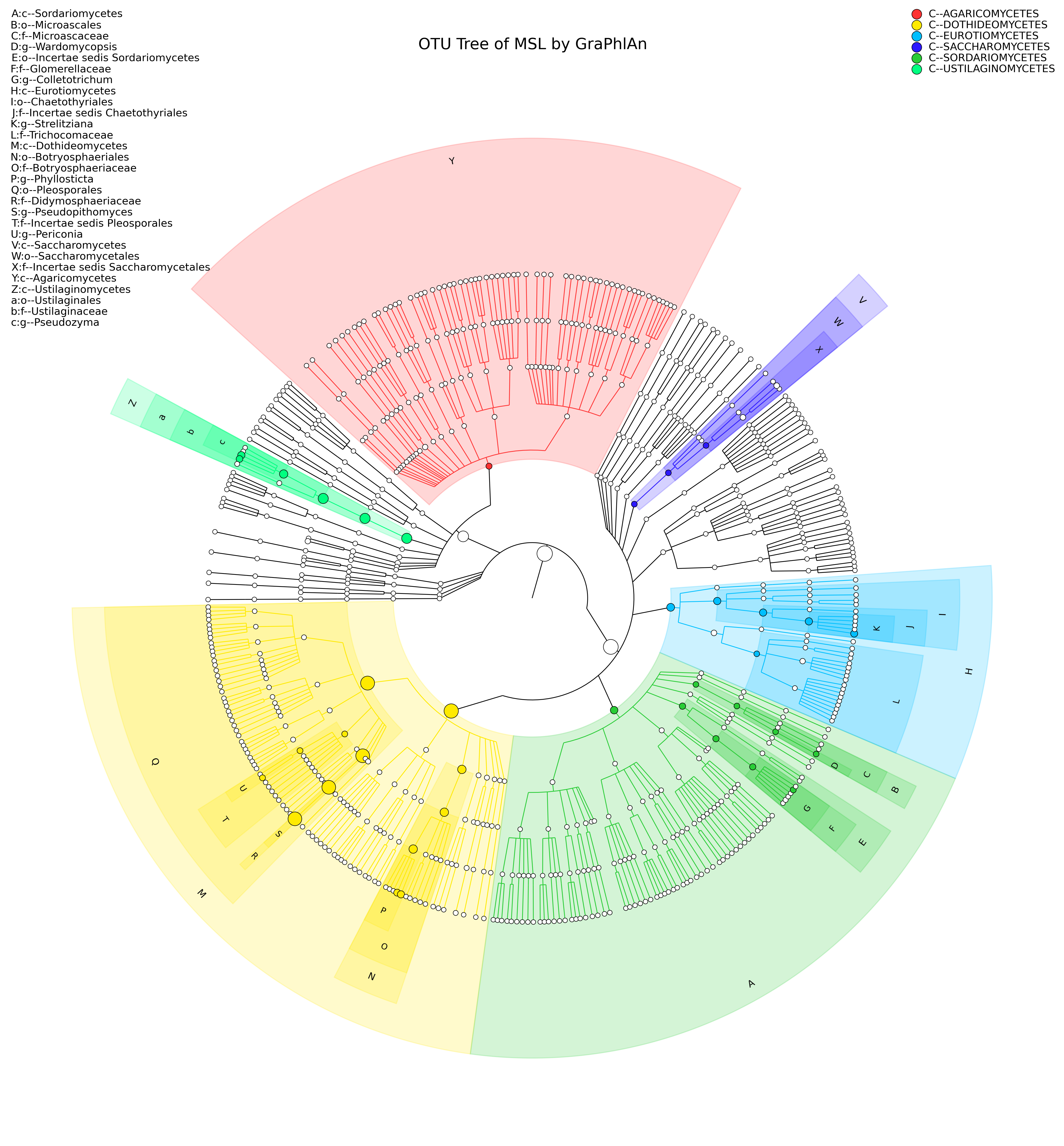

Supplement: Supplementary file 1 [file ijms-19-03421-s001.zip › ijms-348151-supplementary-final check/Supporting imformation-20181026/Figure S10 Hierarchy tree of MSL.png]

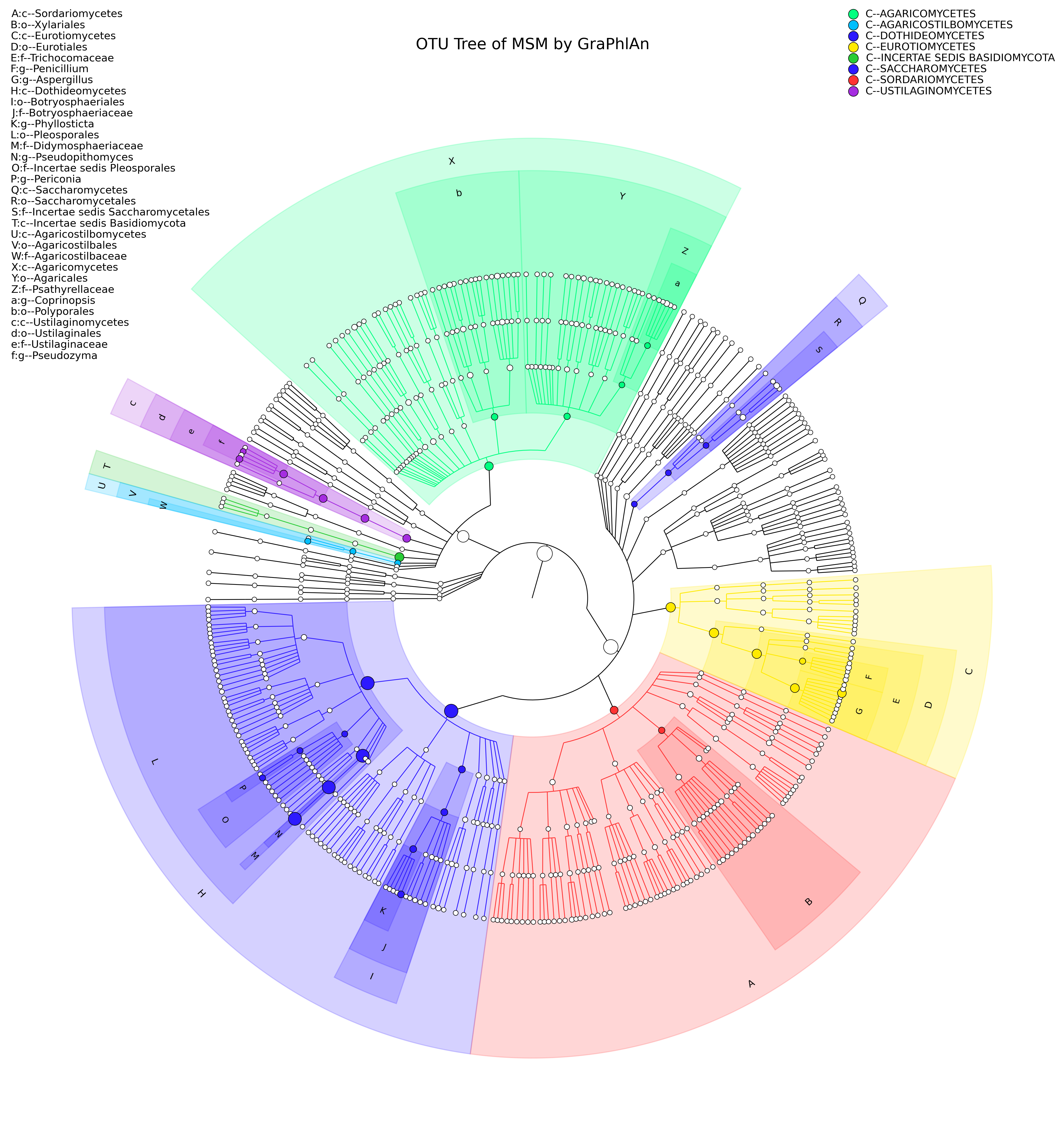

Supplement: Supplementary file 1 [file ijms-19-03421-s001.zip › ijms-348151-supplementary-final check/Supporting imformation-20181026/Figure S11 Hierarchy tree of MSM.png]

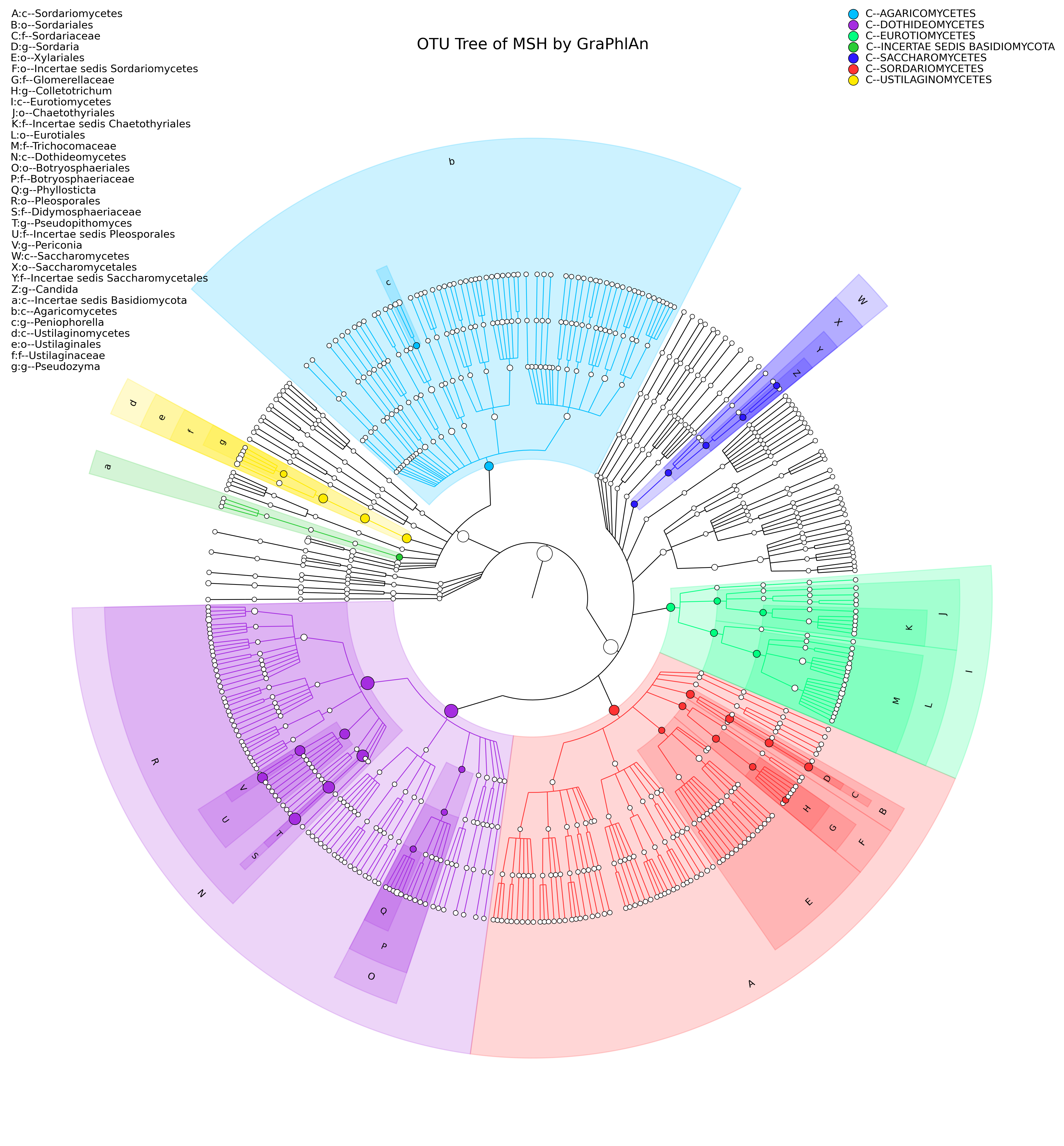

Supplement: Supplementary file 1 [file ijms-19-03421-s001.zip › ijms-348151-supplementary-final check/Supporting imformation-20181026/Figure S12 Hierarchy tree of MSH.png]

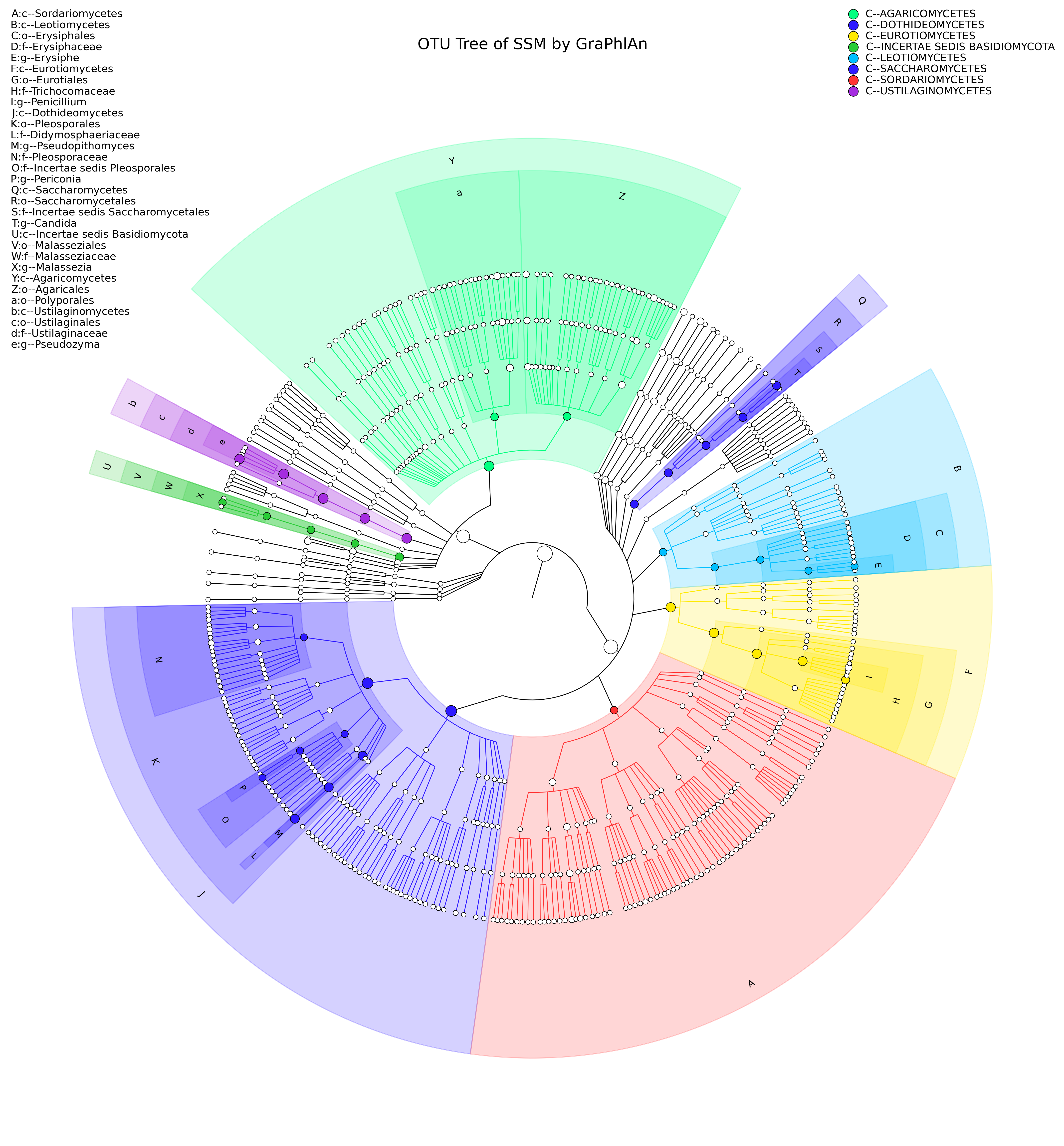

Supplement: Supplementary file 1 [file ijms-19-03421-s001.zip › ijms-348151-supplementary-final check/Supporting imformation-20181026/Figure S2 Hierarchy tree of SSM.png]

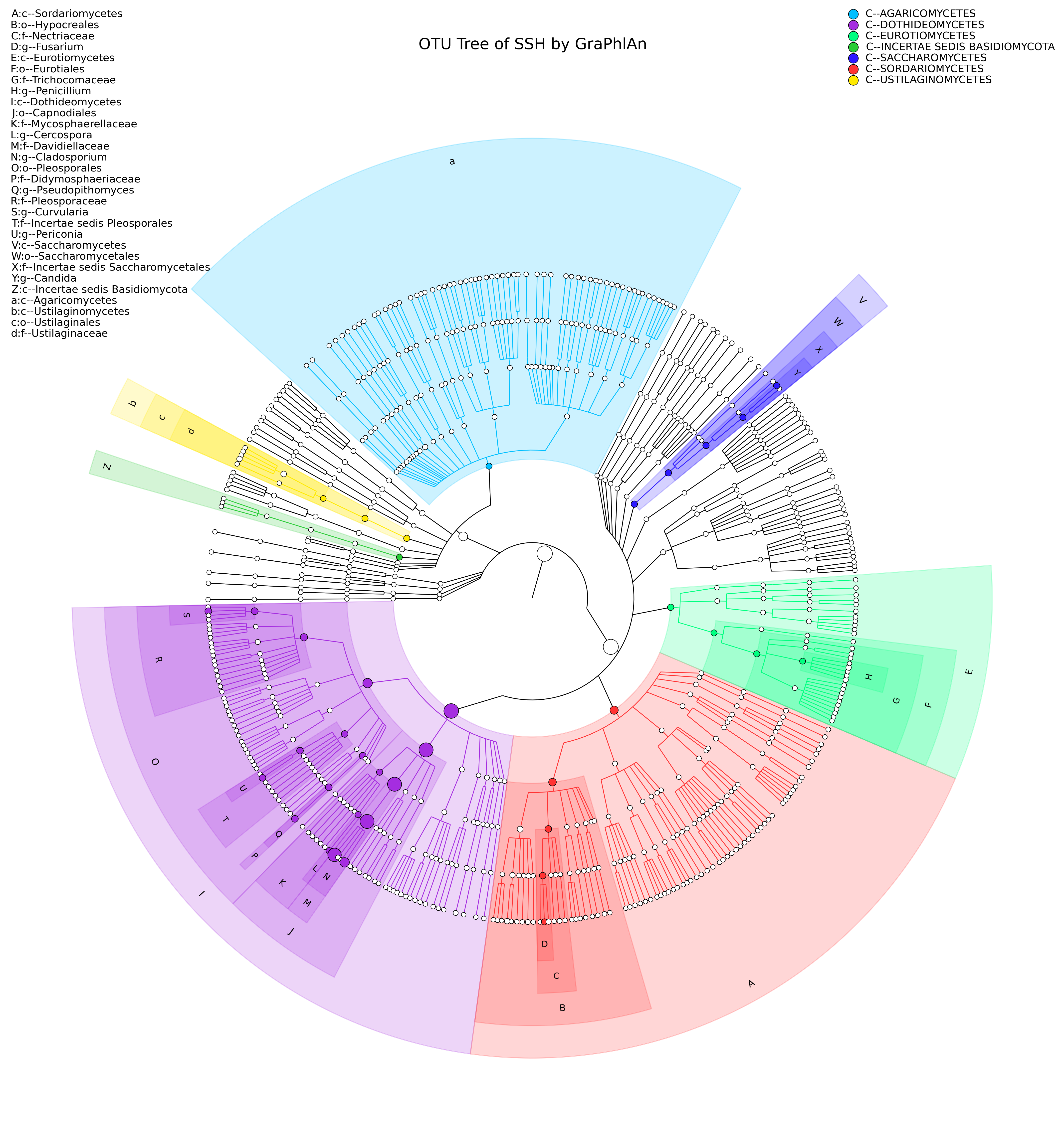

Supplement: Supplementary file 1 [file ijms-19-03421-s001.zip › ijms-348151-supplementary-final check/Supporting imformation-20181026/Figure S3 Hierarchy tree of SSH.png]

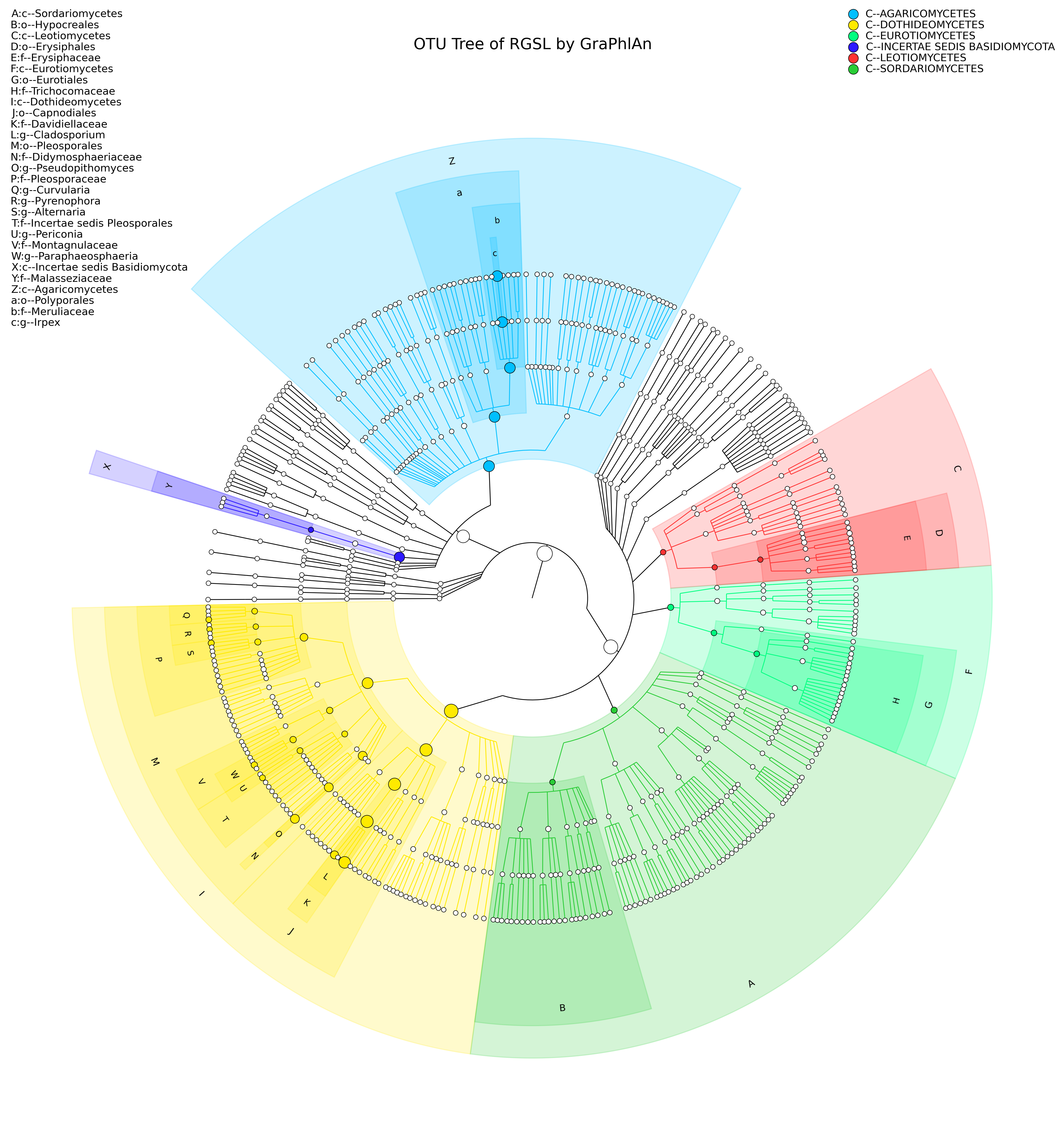

Supplement: Supplementary file 1 [file ijms-19-03421-s001.zip › ijms-348151-supplementary-final check/Supporting imformation-20181026/Figure S4 Hierarchy tree of RGSL.png]

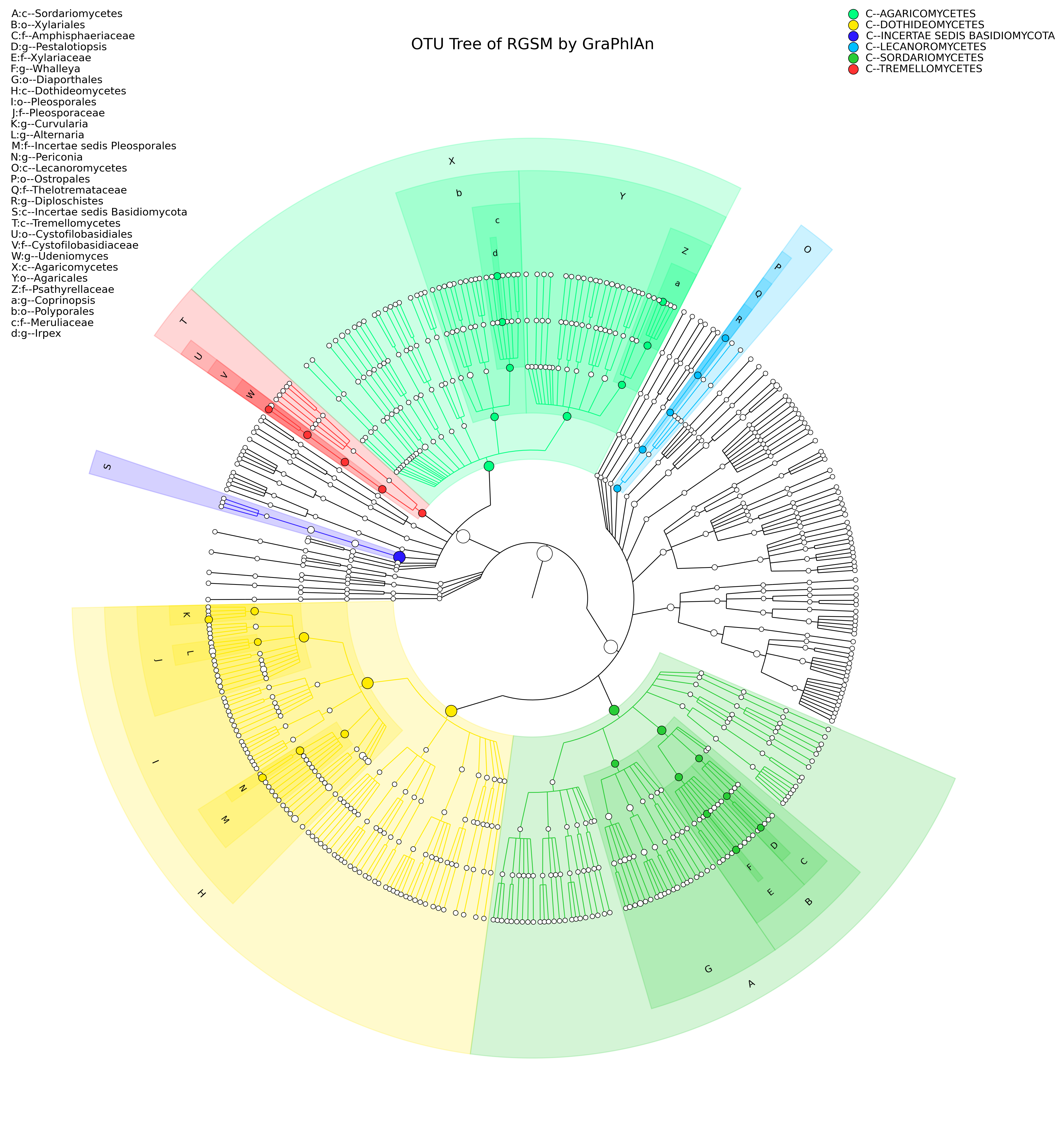

Supplement: Supplementary file 1 [file ijms-19-03421-s001.zip › ijms-348151-supplementary-final check/Supporting imformation-20181026/Figure S5 Hierarchy tree of RGSM.png]

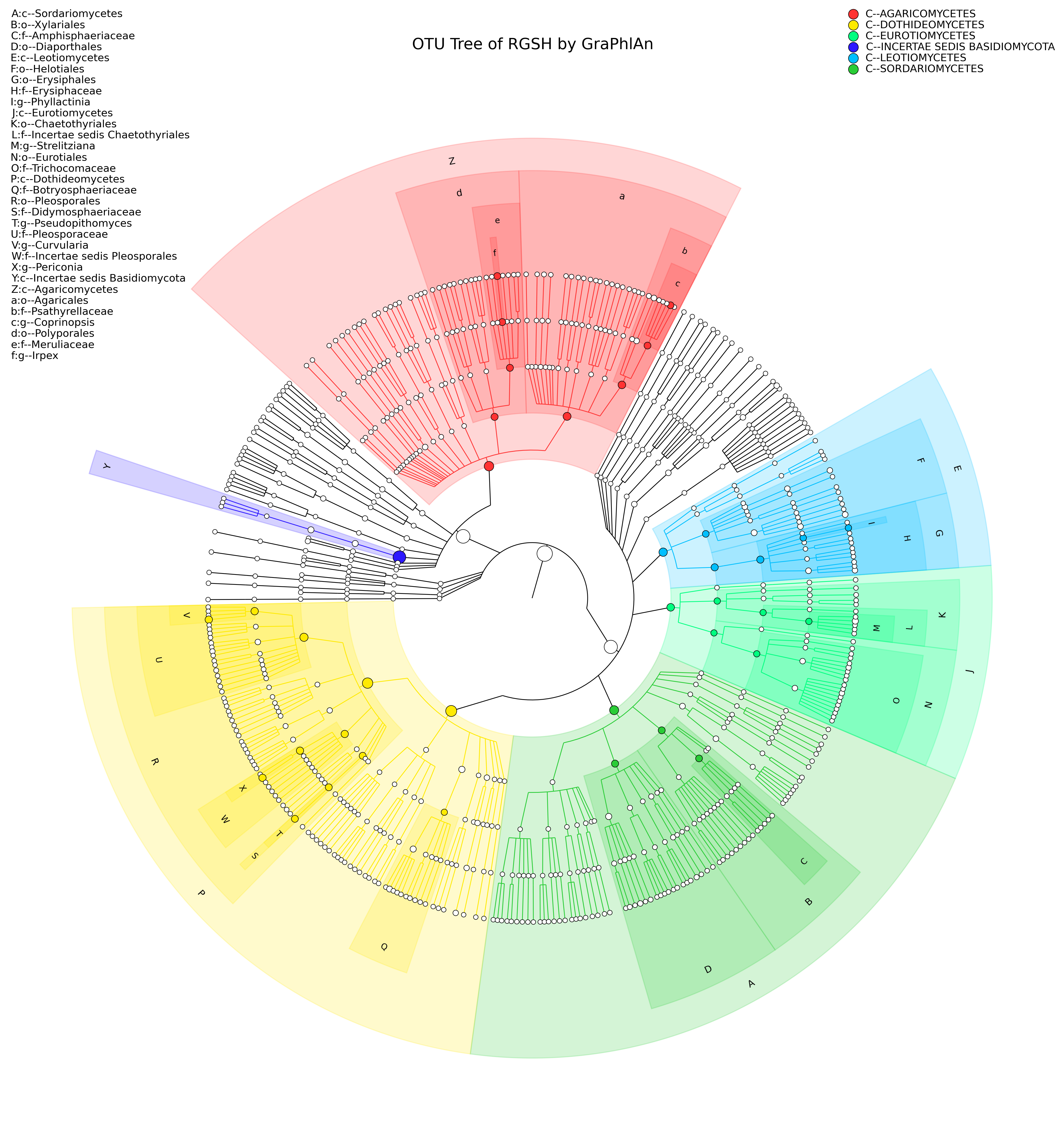

Supplement: Supplementary file 1 [file ijms-19-03421-s001.zip › ijms-348151-supplementary-final check/Supporting imformation-20181026/Figure S6 Hierarchy tree of RGSH.png]

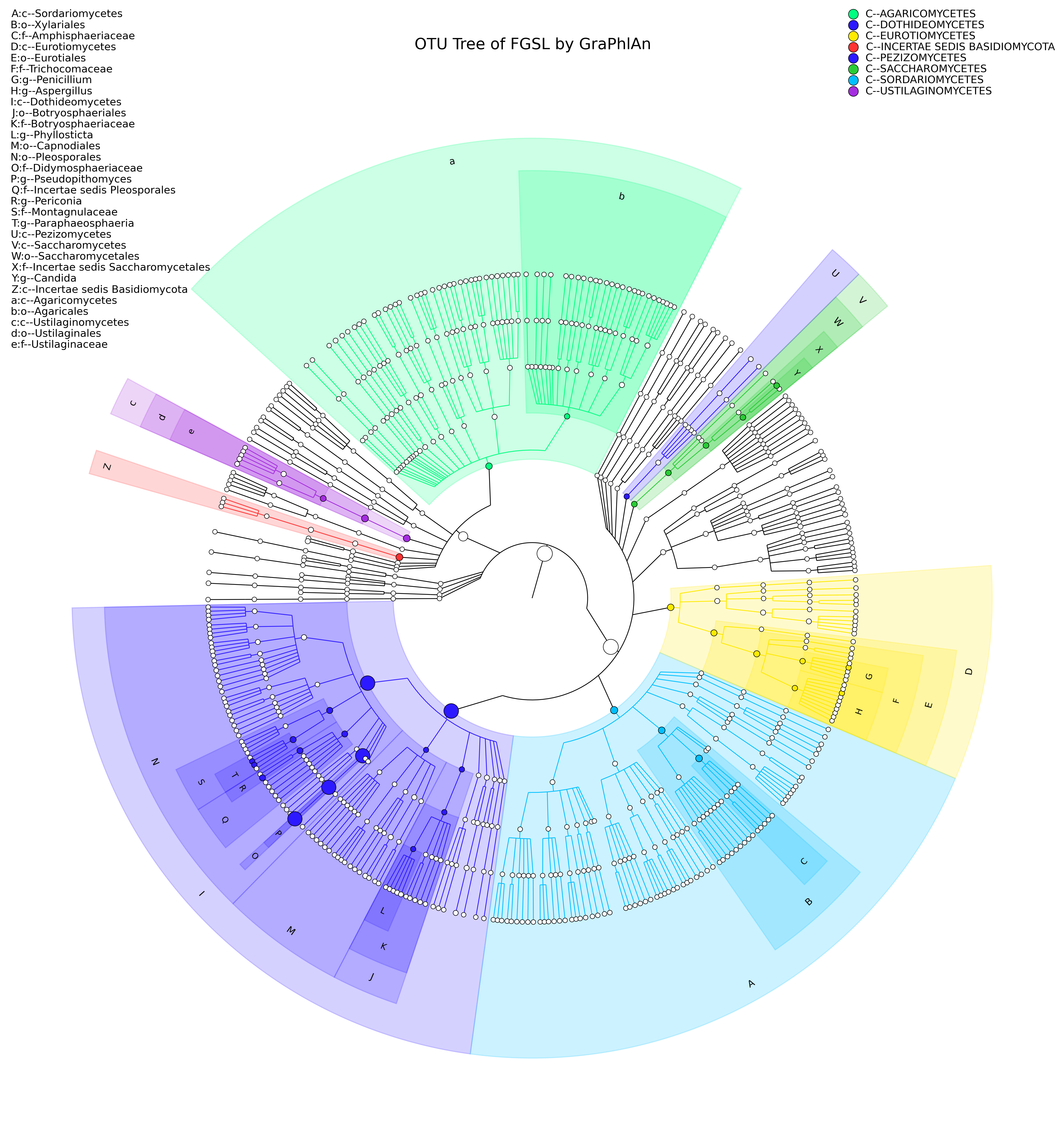

Supplement: Supplementary file 1 [file ijms-19-03421-s001.zip › ijms-348151-supplementary-final check/Supporting imformation-20181026/Figure S7 Hierarchy tree of FGSL.png]

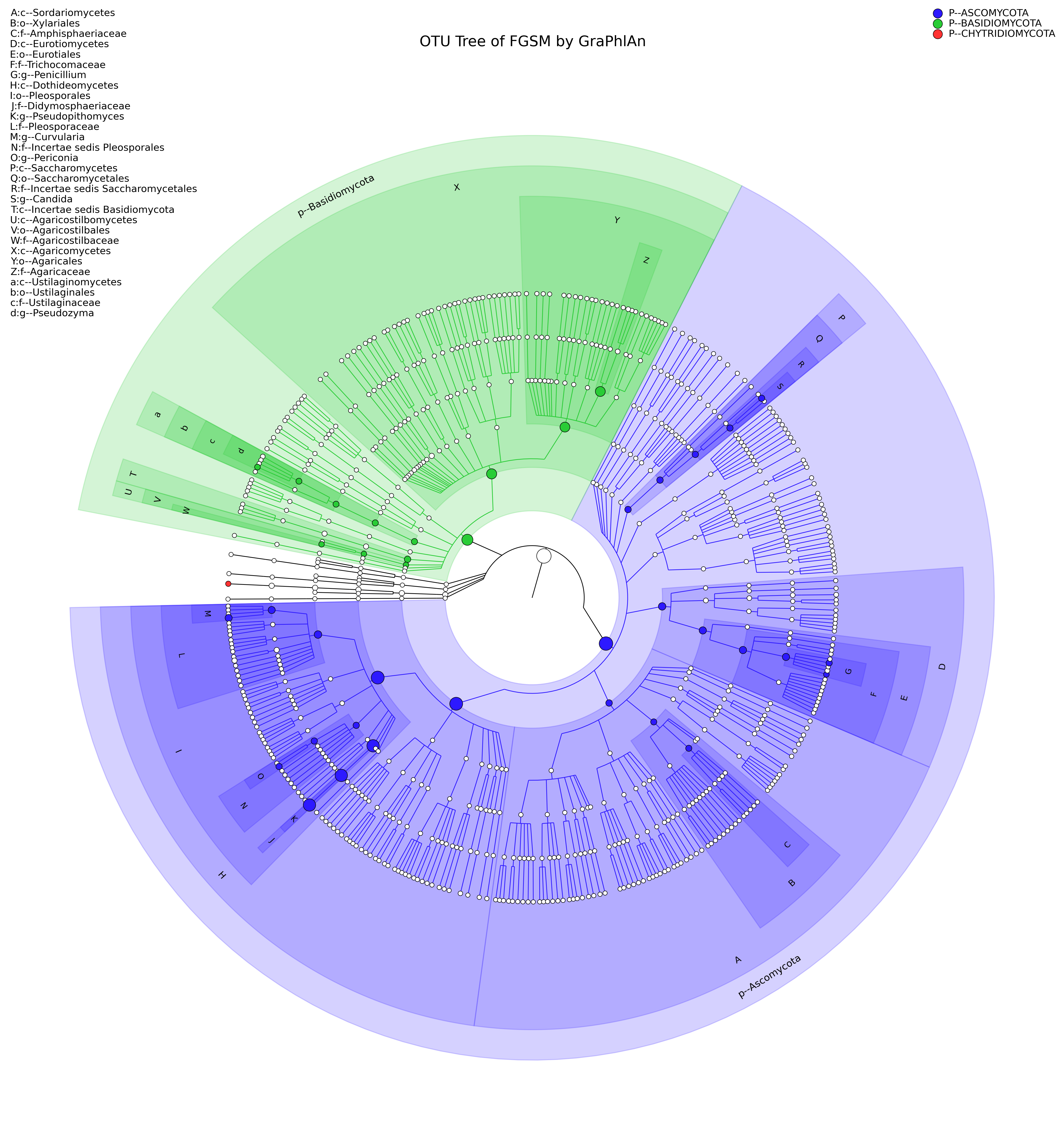

Supplement: Supplementary file 1 [file ijms-19-03421-s001.zip › ijms-348151-supplementary-final check/Supporting imformation-20181026/Figure S8 Hierarchy tree of FGSM.png]

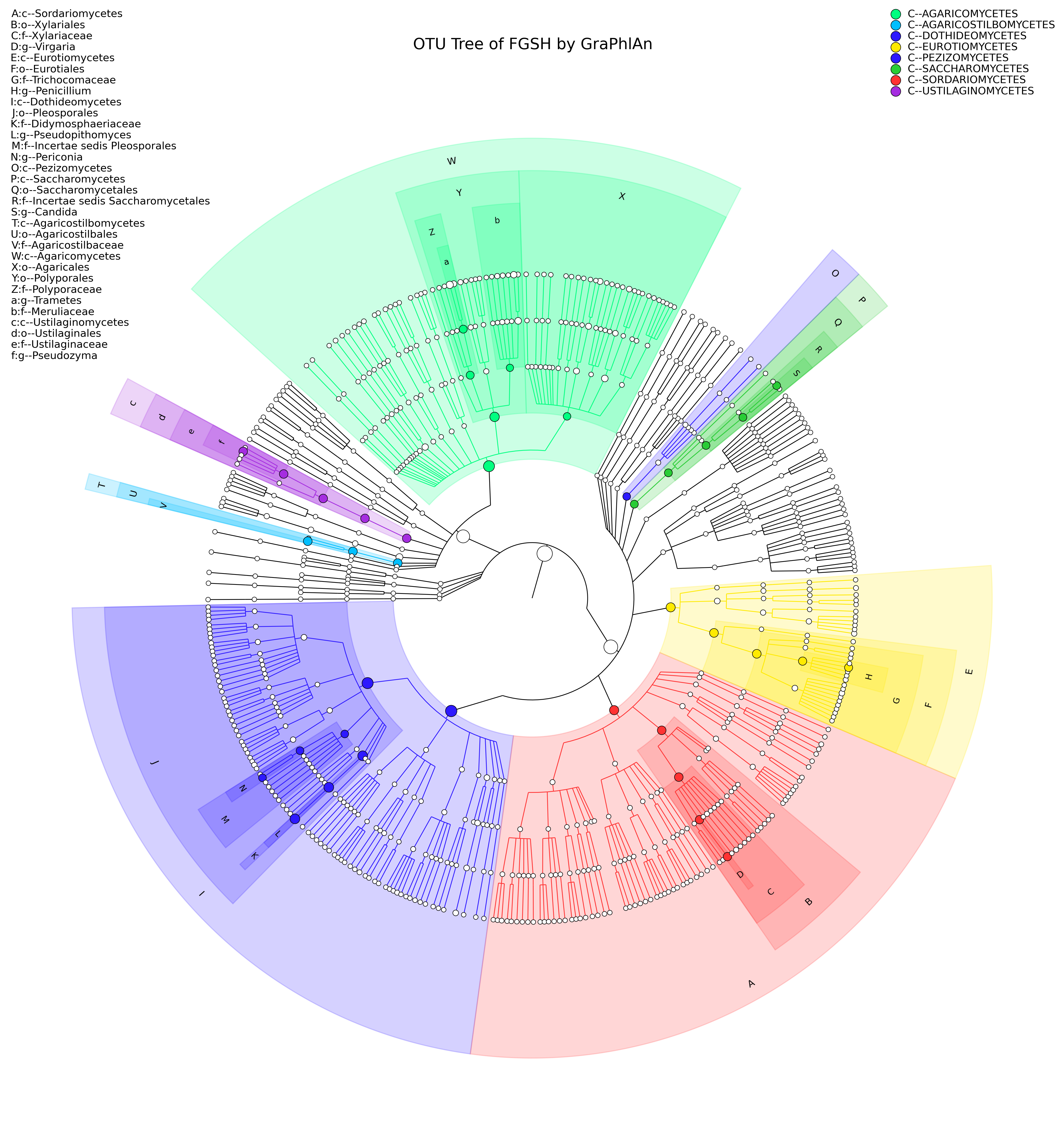

Supplement: Supplementary file 1 [file ijms-19-03421-s001.zip › ijms-348151-supplementary-final check/Supporting imformation-20181026/Figure S9 Hierarchy tree of FGSH.png]
